# Integrated multi-omics analysis reveals the role of nitrogen application in seed storage protein metabolism and improvement of inferior grains in smooth bromegrass

**DOI:** 10.3389/fpls.2025.1605073

**Published:** 2025-09-12

**Authors:** Chengming Ou, Shiqiang Zhao, Zhicheng Jia, Shoujiang Sun, Juan Wang, Chunjiao Mi, Jinyu Shi, Changran Li, Peisheng Mao

**Affiliations:** College of Grassland Science and Technology, China Agricultural University, Key Laboratory of Pratacultural Science, Beijing, Beijing, China

**Keywords:** α-gliadin, nitrogen application, seed vigor, superior and inferior grains, storage protein, smooth bromegrass, multi-omics

## Abstract

This study explored the effects of nitrogen application on superior and inferior grains in smooth bromegrass (*Bromus inermis*) to provide insights for improving seed quality and yield. The study was conducted using a randomized block design with two nitrogen treatments (0 and 200 kg·N·ha^-^¹) during the 2021–2022 growing seasons. Seed dry weight, fresh weight, and storage protein content were measured at multiple stages after anthesis. PacBio full-length transcriptome sequencing generated a comprehensive transcriptome consisting of 124,425 high-quality transcripts, and metabolomic profiling were performed across developmsental stages. Genetic transformation in Arabidopsis was used to validate gene function. Nitrogen application significantly increased seed dry and fresh weights and storage protein content, particularly gliadin and glutelin. Metabolomic and transcriptomic analyses revealed that nitrogen treatment upregulated glutamate and asparagine levels and enhanced nitrogen transport and protein synthesis pathways. Two α-gliadin nitrogen-responsive genes, *BiGli1* and *BiGli2*, were identified. Overexpression of these genes in Arabidopsis confirmed their role in regulating seed size and vigor. This study highlights the critical role of α-gliadin in enhancing seed quality, particularly in promoting the development of inferior grains, offering valuable insights for the development of high-yield seed varieties and the optimization of specialized forage seed production.

## 1 Introduction

Smooth bromegrass (*Bromus inermis* Leyss.) is a perennial, cool-season forage species native to Eurasia and widely cultivated for its high biomass production, palatability, and resilience to environmental stresses such as drought and cold (Saeidnia et al., 2019; Mackiewicz-Walec et al., 2024). Its importance in hay production, grazing, and ecological restoration has led to extensive use; however, low seed yield and poor seed uniformity continue to restrict its adoption and limit further breeding improvements (Ou et al., 2024). In forage seed production, improving seed uniformity and vigor, especially by reducing the developmental gap between superior and inferior grains and enhancing the quality of inferior grains, is essential for successful hay production, livestock grazing, and the commercial seed industry.

A critical factor behind these limitations is the positional difference in seed development within the inflorescence, resulting in “superior” and “inferior” grains due to sequential flowering and filling (Jiang et al., 2003). In rice (*Oryza sativa*), grains developing on upper spikelets and primary branches generally fill better, whereas in wheat (*Triticum aestivum*), grains from the middle and lower spikelets are more robust (Cheng et al., 2007; Kato, 2010, 2020; Mohapatra et al., 2011; Zuo et al., 2012). Inferior grains, particularly under nutrient-limited conditions, compete less effectively for assimilates and are prone to poor development or abortion (Ishimaru et al., 2005; Kamoi et al., 2008). Such deficiencies not only result in low seed vigor and diminished overall quality, but can ultimately limit crop productivity. While these positional effects on grain development have been extensively investigated in cereal crops, they remain poorly understood in forage grasses such as smooth bromegrass.

Seed storage proteins are vital to grain development, yield, and quality, and are typically categorized as albumins, globulins, gliadins, and glutelins (Shewry and Halford, 2002). Their synthesis is tightly regulated by genetic and environmental factors. For instance, modifying genes such as *RAG2*, *Glb-1*, or γ-gliadins can significantly alter grain size and storage protein content (Qu and Takaiwa, 2004; Kurokawa et al., 2014; Liu et al., 2023). Among environmental inputs, nitrogen availability is one of the most effective interventions (Govindasamy et al., 2023); it regulates the expression of protein synthesis pathways and thus boosts storage protein content (Barneix, 2007; Yu et al., 2017). For example, wheat grain protein content can nearly double under high nitrogen management (Godfrey et al., 2010; Zhang et al., 2016). Similarly, Türk et al (Turk et al., 2018)reported that the yields and quality of smooth bromegrass forage can be optimized when nitrogen is supplied at 120 to 160 kg·ha^-^¹.

Despite the recognized importance of nitrogen, little is known about how its application differentially affects protein synthesis and accumulation in superior versus inferior grains of smooth bromegrass. This gap limits our capacity to improve seed uniformity and quality through targeted breeding and agronomic strategies. Therefore, understanding the molecular mechanisms of nitrogen-regulated protein metabolism in distinct grain types will provide a theoretical foundation for future improvement in forage seed production.

Advances in transcriptomics, metabolomics, proteomics, and molecular biology have greatly facilitated the discovery of key genes and pathways involved in seed development (Kovacik et al., 2024; Yin et al., 2024). High-quality genome assemblies are essential for omics-based analyses, yet genomic research in forage crops has lagged far behind that in major cereals such as rice and wheat, constraining molecular breeding efforts for these species. While chromosome-scale assemblies are now available for several important forage crops, including alfalfa (*Medicago sativa*) (Chen et al., 2020), oat (*Avena sativa*) (Kamal et al., 2022), orchardgrass (*Dactylis glomerata*) (Huang et al., 2020), and awnless cleistogenes (*Cleistogenes songorica*) (Zhang et al., 2021), smooth bromegrass (*Bromus inermis*), with its complex genetic background and large genome size, still lacks a reference genome. Consequently, transcriptomic studies in this species typically rely on *de novo* assembly or reference genomes from related model crops (Zhou et al., 2020; Gong et al., 2024).

Recent advances in sequencing platforms, particularly PacBio Single Molecule Real-Time (SMRT) sequencing, enable the direct generation of full-length transcript isoforms with long reads and high accuracy, providing powerful advantages for analyzing complex transcriptomes (Abdel-Ghany et al., 2016; Wang et al., 2016; Luo et al., 2019). This technology has been successfully applied to investigate stress responses and developmental processes in various crops.

In this study, we integrated PacBio SMRT and Illumina short-read sequencing with metabolomic profiling to examine the effects of nitrogen application on protein metabolism in superior and inferior grains of smooth bromegrass across key developmental stages. Our goals were to elucidate the major regulatory networks and metabolic pathways underlying nitrogen-induced changes in storage protein synthesis, identify candidate nitrogen-responsive genes, and provide molecular insights to improve seed quality while narrowing the gap between superior and inferior grains.

## 2 Materials and methods

### 2.1 Plant materials and experimental design

The experiment was conducted over two growing seasons (2021–2022) at the Forage Seed Production Experimental Base of China Agricultural University, located at Yuershan Ranch, Chengde City, Hebei Province, China (41°44′ N, 116°8′ E; elevation 1455 m). The site features a semi-arid continental monsoon climate with an 85-day frost-free period. Soil properties included 27.63 g·kg^-^¹ organic matter, 20.58 mg·kg^-^¹ available nitrogen, 10.40 mg·kg^-^¹ available phosphorus, and 53.25 mg·kg^-^¹ available potassium. A randomized complete block design was employed, comprising four blocks and two nitrogen treatments: 0 kg·N·ha^-^¹ (control, CK) and 200 kg·N·ha^-^¹ (N), applied as urea (46% N). The plant material used was the cultivar ‘Yuanye’ of smooth bromegrass, purchased from Beijing Zhengdao Ecological Technology Co., Ltd. The seeds were produced in Canada, with a purity of 97.2% and a germination rate of 80%. Smooth bromegrass seeds were sown on 8 July 2020 at a row spacing of 45 cm in plots measuring 4 m × 5 m, with a theoretical sowing rate of 30 kg·ha^-^¹. Nitrogen fertilizer was applied in May of both 2021 and 2022 after spring regreening, with the application rate determined according to local agronomic recommendations and prior field research.

### 2.2 Analysis on physiological index

During the 2021 and 2022 flowering periods of smooth bromegrass, fertile tillers flowering on the same day were tagged and sampled at 10, 16, 23, and 30 days after anthesis (DAA). Spikelets from the inflorescence’s middle portion were collected to differentiate grain positions (GP): the first and second grains from the spikelet base were classified as superior grains (SG), and the third to fifth as inferior grains (IG)[3]. The field sampling method, including the positions of superior and inferior grains within the spikelet and their appearance at different developmental stages, is illustrated in **Figure S1**. For each treatment, 160 uniform seeds were weighed fresh using an analytical balance (0.001 g precision), then oven-dried at 130°C for 2 h to record dry weight. Next, 0.3 g of dried seeds per treatment (four replicates) was ground, and nitrogen content was measured via the Kjeldahl method, with protein content calculated using a 6.25 conversion factor. Seeds harvested at 30 DAA were air-dried at 20–25°C for 2–3 days; then, 1.000 g of air-dried seeds underwent sequential extraction of albumin, globulin, gliadin, and glutelin with continuous shaking (Bradford, 1976; Iida et al., 1993). These protein fractions were quantified using the Coomassie Brilliant Blue method.

### 2.3 Samples for PacBio sequencing and illumina sequencing

Short-read RNA sequencing was performed on the Illumina HiSeq platform (paired-end, 150 bp), and full-length transcriptome sequencing was performed on the PacBio Sequel system. For PacBio sequencing, samples comprised seeds, stems, leaves, roots, and shoots of smooth bromegrass. For seed samples, five grains from each of the three developmental stages (16, 23, and 30 DAA) were pooled together. At 16 DAA, stems and leaves were harvested from five plants with uniform growth. Seeds were germinated in a growth chamber (16 h dark/8 h light, 15/25°C) for 14 days; subsequently, roots and shoots were collected from 40 uniformly sized seedlings. All samples were flash-frozen in liquid nitrogen and stored at -80°C. For Illumina sequencing, seed samples from 16, 23, and 30 DAA were categorized into 12 treatments (three time points × two grain positions [superior: SG; inferior: IG] × two conditions [control: CK; nitrogen-treated: N]), labeled as CS16, CI16, NS16, NI16, CS23, CI23, NS23, NI23, CS30, CI30, NS30, and NI30. Each treatment included eight uniform seeds with three replicates.

### 2.4 RNA preparation, library construction, sequencing and Iso-Seq data processing

Total RNA was extracted from plant samples using the RNAprep Pure Plant Kit (Tiangen, Beijing, China) following the manufacturer’s protocol. RNA quantity and purity were measured with a NanoDrop 2000 spectrophotometer (Thermo Fisher Scientific, Wilmington, DE, USA), and integrity was assessed using an RNA Nano 6000 Assay Kit on an Agilent Bioanalyzer 2100 (Agilent Technologies, Santa Clara, CA, USA). For PacBio Iso-Seq, high-quality RNA from seeds, stems, leaves, roots, and shoots was pooled at a 2:1:1:1:1 ratio for full-length cDNA synthesis (NEBNext^®^ Single Cell/Low Input cDNA Synthesis & Amplification Module, New England Biolabs, Ipswich, MA, USA) and SMRTbell library construction (SMRTbell Express Template Prep Kit 2.0, Pacific Biosciences, Menlo Park, CA, USA). For Illumina sequencing, eukaryotic mRNA was enriched with Oligo(dT) magnetic beads, fragmented, reverse-transcribed, and converted into sequencing libraries following the manufacturer’s standard workflow. Libraries were purified with AMPure XP beads (Beckman Coulter, Brea, CA, USA) and quality-checked on an Agilent Bioanalyzer 2100 before sequencing.

### 2.5 Assembly, annotation of SMRT and illumina sequence assembly

Iso-Seq sequencing was performed on the PacBio Sequel system. Raw data were filtered to exclude fragments <50 bp and sequences with accuracy <0.90. Adapter sequences were removed to obtain subreads, discarding those <50 bp to produce clean data. Clean reads with ≥3 full passes and accuracy >0.90 were processed into circular consensus sequences (CCS). CCS with intact 5’/3’ primers and polyA tails were classified as full-length non-chimeric (FLNC) sequences. The IsoSeq module in SMRTLink clustered similar FLNC sequences into consensus isoforms, generating high-quality (HQ, >99% accuracy) and low-quality (LQ) transcripts. CD-HIT removed redundant sequences, yielding non-redundant full-length transcripts, which were annotated against public databases (NR, SwissProt, COG, KOG, Pfam, GO, KEGG) using *DIAMOND*.

These transcripts served as a reference for aligning second-generation sequencing data. STAR (v2.7.10a; Dobin et al., 2013) mapped clean reads to the transcripts, and RSEM (v1.3.3; Li and Dewey, 2011)quantified expression as FPKM (Fragments Per Kilobase of transcript per Million mapped fragments). RSEM calculated gene read counts, and *DESeq2* (v1.38.3; Love et al., 2014) identified differentially expressed genes (DEGs) across replicated samples (Fold Change ≥1.50, *P* value <0.01). DEGs were annotated using the full-length transcript annotations, followed by KEGG and GO enrichment analyses with R packages “*clusterProfiler* (v4.6.2; Wu et al., 2021)” and “*ggplot2* (v3.4.1; Ginestet, 2011)”.

DEG analysis of 12 treated samples (CS16, CI16, NS16, NI16, CS23, CI23, NS23, NI23, CS30, CI30, NS30, NI30) identified 36,929 DEGs. Genes with *FPKM <*0.1 and MAD >1 across all samples were excluded, leaving 18,853 DEGs for WGCNA analysis (Weighted correlation network analysis). In R, with a soft threshold of beta=9, an adjacency matrix clustered DEGs into color-coded modules based on expression patterns. Modules responsive to nitrogen treatment were identified, and their genes were functionally classified.

### 2.6 Untargeted metabolomic analysis

A 50 mg sample of smooth bromegrass seeds, ground in liquid nitrogen, was mixed with 1000 μL of extraction solvent (methanol:acetonitrile:water, 2:2:1, v/v, 20 mg/L internal standard) and vortexed for 30 s. Steel beads were added, and the mixture was ground at 45 Hz for 10 min, ultrasonicated in an ice-water bath for 10 min, and held at -20°C for 1 h. After centrifugation at 12,000 rpm and 4°C for 15 min, 500 μL of supernatant was dried in a vacuum concentrator and reconstituted in 160 μL of acetonitrile:water (1:1, v/v). This was vortexed for 30 s, ultrasonicated for 10 min, and centrifuged again at 12,000 rpm and 4°C for 15 min. Then, 120 μL of supernatant was collected in a 2 mL vial, with 10 μL from each sample pooled as a QC sample. Analysis used a Waters Acquity I-Class PLUS UPLC coupled to a Waters Xevo G2-XS QTOF mass spectrometer. Raw data, acquired with *MassLynx* V4.2, were processed in Progenesis QI for peak extraction and alignment. Metabolites were identified using METLIN, public databases, and Biomaker Technologies’library, with fragment matching. Mass deviation limits were 100 ppm for precursor ions and 50 ppm for fragment ions.

### 2.7 Generation of the overexpression Arabidopsis plants

Based on Section 2.5, *BiGli1* and *BiGli2* were selected as target genes. Their ORFs were PCR-amplified from smooth bromegrass seed cDNA (primers in **Supplementary Table S1**), cloned into *PC-GW-Hyg-eGFP* via *pCE-Zero*, and introduced into *Agrobacterium tumefaciens* GV3101. Arabidopsis was transformed using the floral dip method. Transformants were selected on MS medium with 50 µg/mL hygromycin, and T3 homozygous lines were confirmed for resistance.

The transcript levels of *BiGli1* and *BiGli2* in T3 homozygous lines were determined by RT-qPCR using *UBQ10* as the reference gene (primers in **Supplementary Table S1**), with seeds collected 3 days after germination. Based on relative expression, three representative OE lines for each gene (*BiGli1* OE2, OE4, OE6; *BiGli2* OE1, OE3, OE6) were selected for seed propagation and subsequent experiments. Protein accumulation in these lines was confirmed by Western blot analysis using anti-GFP antibody (Bio-swamp, TAG10014, 1:4000); β-actin (Bio-swamp, MAB48206, 1:10000) was used as a loading control, also using seeds collected 3 days after germination.

### 2.8 Phenotypic analysis of overexpression Arabidopsis seeds

T3 overexpression Arabidopsis and Columbia (Col) seeds, harvested simultaneously, were photographed using a stereomicroscope. Seed surface area, length, and width were measured with *ImageJ* from 30 randomly selected seeds per line.

### 2.9 CDT and phenotype analysis of overexpression Arabidopsis seeds

Homozygous overexpression Arabidopsis seeds (1 g) were desiccated with silica gel and aged using the controlled deterioration treatment (CDT) method described by Chen et al (Chen et al., 2012). Seeds were placed in a 4-cm Petri dish within a desiccator at 85% humidity (saturated KCl) and equilibrated at 20°C for 3 days. Aging proceeded at 40°C and 85% relative humidity for 3 days, followed by equilibration with saturated MgCl_2_ for 3 days. After disinfection and vernalization, aged seeds were sown on 0.5× MS agar plates for phenotypic analysis.

### 2.10 Statistical analysis

Seed traits under two nitrogen levels were compared using Student’s t-test (*P* < 0.05), with analyses performed in the R package *agricolae v1.3-7*. Line plots were generated using *GraphPad Prism 8.0*.

## 3 Results

### 3.1 Effect of nitrogen application on dry and fresh weights of superior and inferior grains

**Figure 1:**
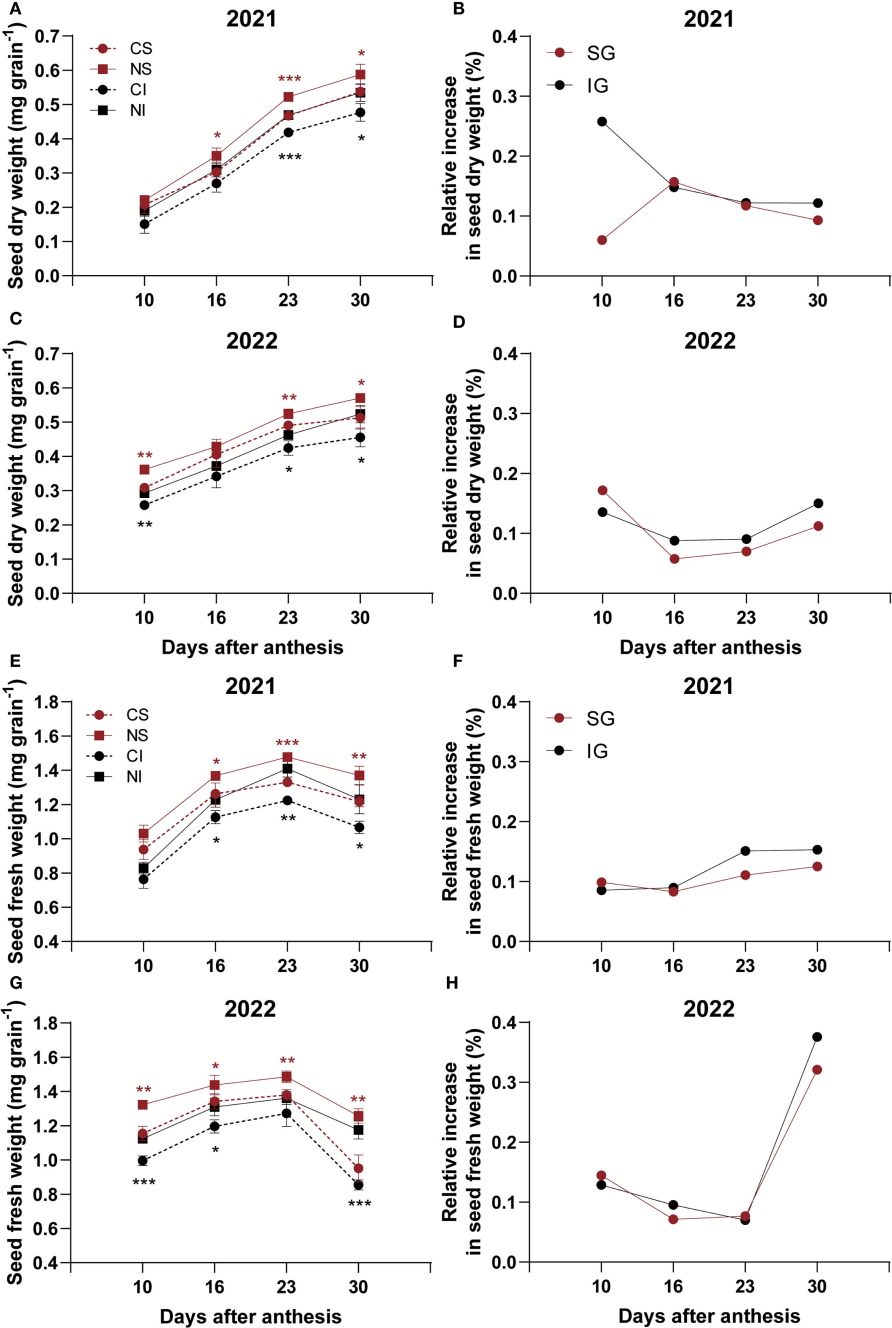
Effects of nitrogen on dry and fresh weights of superior (SG) and inferior (IG) grains in smooth bromegrass (2021–2022). **(A, C)** Seed dry weight in 2021 and 2022. **(B, D)** Relative increase in seed dry weight in 2021 and 2022. **(E, G)** Seed fresh weight in 2021 and 2022. **(F, H)** Relative increase in seed fresh weight in 2021 and 2022. CS: SG without nitrogen application; NS: SG with nitrogen application; CI: IG without nitrogen application; NI: IG with nitrogen application. Asterisks denote significant differences between control and nitrogen treatments (* *P* < 0.05; ** *P* < 0.01; *** *P* < 0.001). Black indicates IG; red indicates SG.

In 2021, nitrogen application significantly (*P* < 0.05) increased dry weights of superior grains (SG) at 16, 23, and 30 DAA and inferior grains (IG) at 23 and 30 DAA, with the most notable increase in IG at 30 DAA reaching 24.2% (**Figure 1A**). At 10 and 30 DAA, IG showed a greater relative dry weight increase than SG (**Figure 1B**). In 2022, nitrogen significantly (*P* < 0.05) increased SG dry weights at 10, 23, and 30 DAA and IG dry weights at 10, 23, and 30 DAA (**Figure 1C**). Relative dry weight increases were higher in SG at 10 DAA but greater in IG at 16, 23, and 30 DAA (**Figure 1D**).

For fresh weights in 2021, nitrogen significantly (*P* < 0.05) increased both SG and IG at 16, 23, and 30 DAA (**Figure 1E**), with IG showing greater relative increases at 23 and 30 DAA (**Figure 1F**). In 2022, nitrogen significantly (*P* < 0.05) increased SG fresh weights across all measured time points and IG fresh weights at 10, 16, and 30 DAA, leading to a substantial 25.5% increase in the fresh weight of IG at 30 DAA (**Figure 1G**). Relative fresh weight increases were greater for IG at 16 and 30 DAA (**Figure 1H**).

### 3.2 Nitrogen effects on seed vigor and protein content

**Figure 2:**
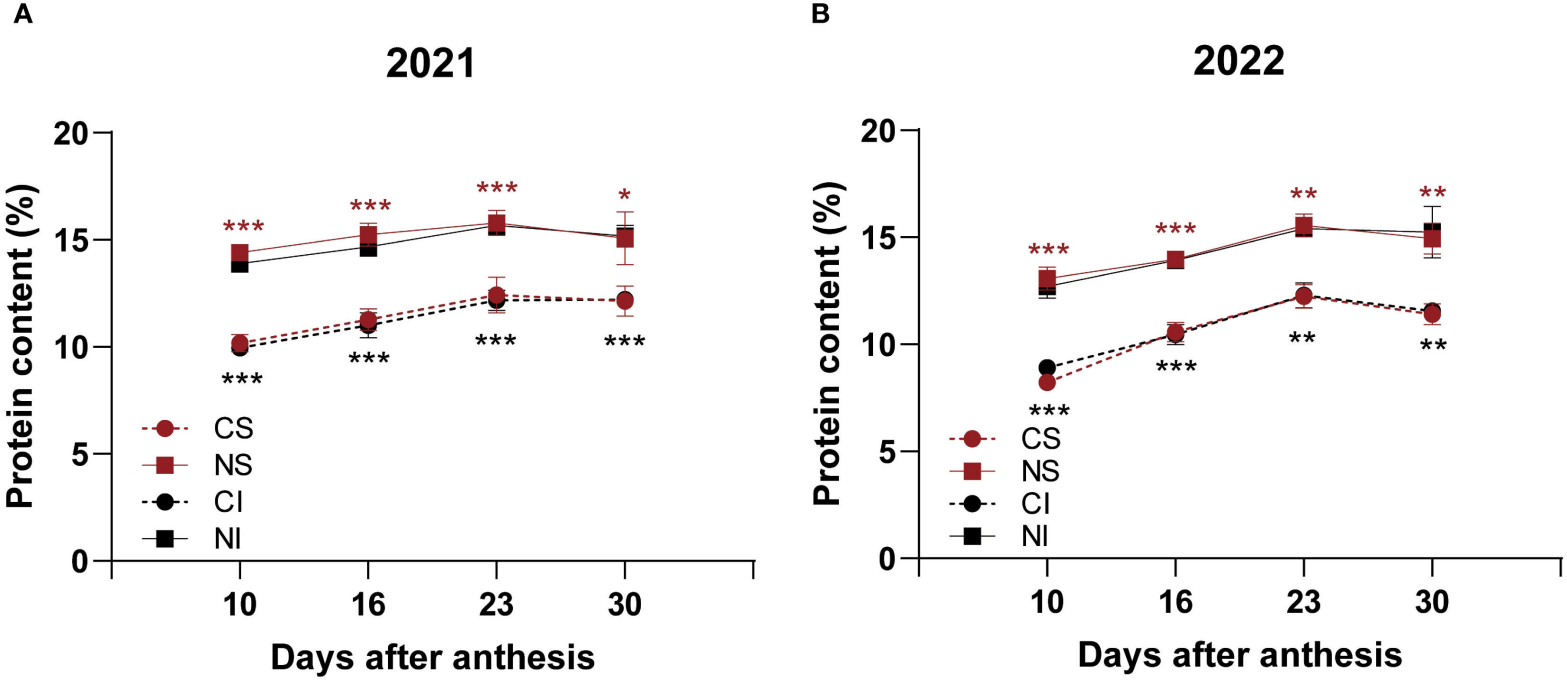
Effects of nitrogen on protein content of SG and IG in smooth bromegrass (2021-2022). **(A)** Protein content in 2021. **(B)** Protein content in 2022. CS: SG without nitrogen application; NS: SG with nitrogen application; CI: IG without nitrogen application; NI: IG with nitrogen application. Asterisks denote significant differences between control and nitrogen treatments (* *P* < 0.05; ** *P* < 0.01; *** *P* < 0.001). Black indicates IG; red indicates SG.

Nitrogen application significantly (*P <* 0.05) increased the total seed protein content in both SG and IG at all developmental stages (10, 16, 23, and 30 DAA) across both years, with relative increases ranging from 17.3% to as high as 60.2% (**Figure 2**, **Supplementary Table S2**). The protein components—albumin, globulin, gliadin, and glutelin—were quantified in mature seeds harvested at 30 DAA (**Table 1**). In 2021, nitrogen application resulted in significant (*P* < 0.05) increases in globulin content in IG (by 28.7%), glutelin content in IG (by 25.8%), and gliadin content in both SG (by 17.4%) and IG (by 20.0%). In 2022, nitrogen significantly *(P* < 0.05) increased glutelin content in both SG (by 18.3%) and IG (by 20.9%), and gliadin content in IG (by 24.3%).

**Table 1:** Effects of nitrogen on protein components of SG and IG in smooth bromegrass (2021-2022).

Year	Treatment	Albumin (mg/g)	Globulin (mg/g)	Gliadin (mg/g)	Glutelin (mg/g)
2021	CI	10.00 ± 0.88 a	7.92 ± 0.49 b	9.06 ± 0.23 b	26.25 ± 0.63 b
	CS	10.55 ± 0.91 a	8.38 ± 0.13 b	9.50 ± 0.17 b	25.63 ± 0.75 b
	NI	12.79 ± 0.95 a	10.19 ± 0.83 a	10.87 ± 0.12 a	33.02 ± 1.23 a
	NS	10.35 ± 0.88 a	9.52 ± 0.53 ab	11.15 ± 0.26 a	29.46 ± 1.94 ab
2022	CI	11.58 ± 0.58 a	8.04 ± 0.39 a	8.90 ± 0.25 b	27.73 ± 0.62 b
	CS	9.90 ± 0.72 a	7.51 ± 0.60 a	8.89 ± 0.06 b	26.82 ± 0.77 b
	NI	12.32 ± 1.21 a	8.79 ± 0.88 a	11.06 ± 1.02 a	33.52 ± 0.76 a
	NS	11.12 ± 0.71 a	8.84 ± 0.51 a	9.75 ± 0.06 ab	31.73 ± 1.74 a

CS: SG without nitrogen application; NS: SG with nitrogen application; CI: IG without nitrogen application; NI: IG with nitrogen application. Means within the same year and column followed by different lowercase letters differ significantly at *P* < 0.05.

### 3.3 Metabolite responses to nitrogen in superior and inferior grain

Metabolomic analysis compared nitrogen effects on SG and IG across six conditions. In SG, nitrogen-treated (NS) versus untreated (CS) samples at 16, 23, and 30 DAA showed 311, 350, and 313 differential metabolites, respectively, with 13 shared across stages (**Supplementary Figure S2A**). In IG, CI_vs_NI comparisons at the same time points revealed 405, 433, and 195 differential metabolites, with 16 common (**Supplementary Figure S2B**).

**Figure 3:**
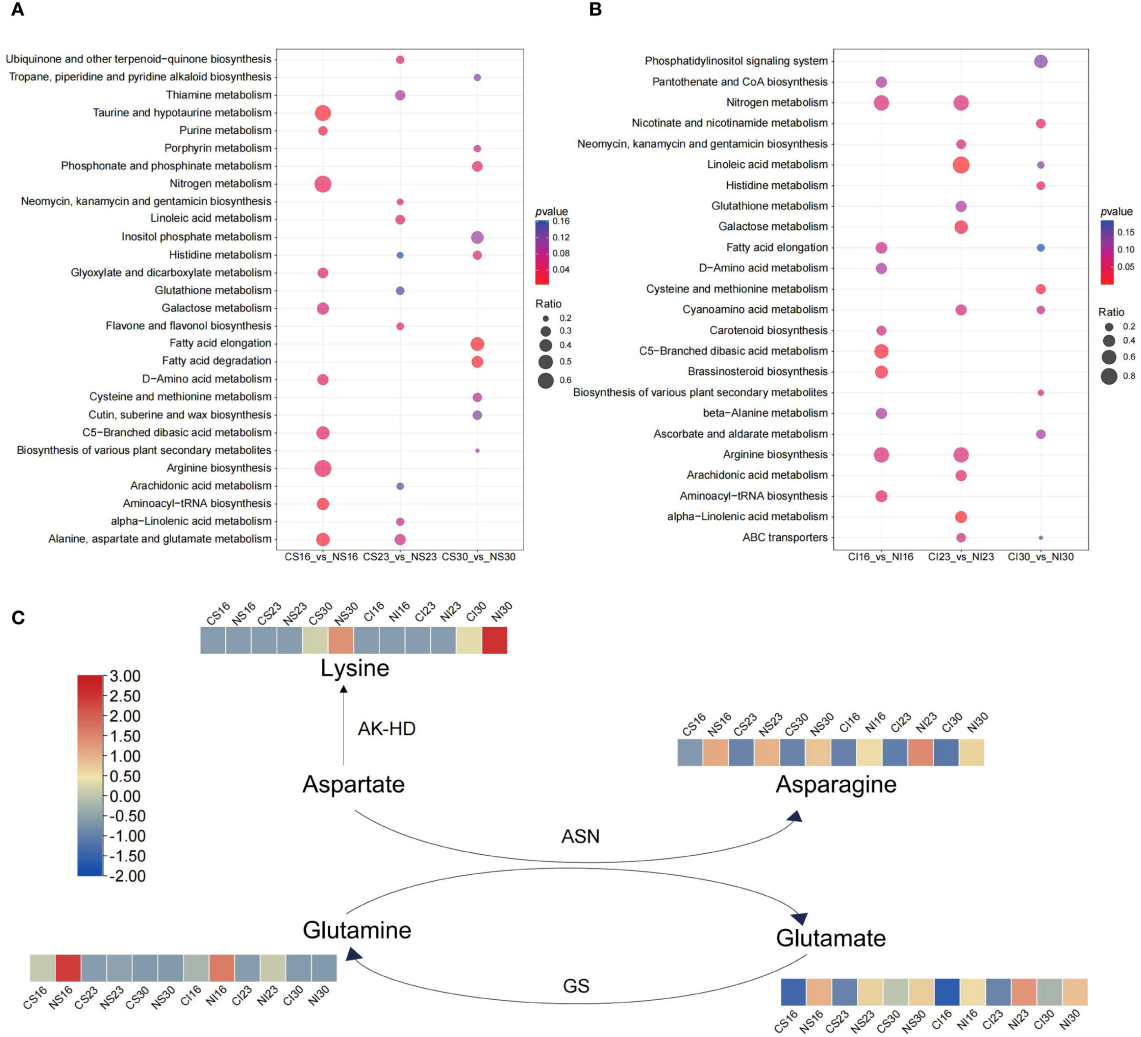
KEGG pathway enrichment analysis for SG **(A)** and IG **(B)**, and variation patterns of differential metabolite contents linked to glutamate and asparagine metabolism **(C)**.

Pathway shifts differed by grain type. In SG, nitrogen enriched taurine and amino acid metabolism at 16 DAA, linoleic acid and flavonoid biosynthesis at 23 DAA, and fatty acid and histidine metabolism at 30 DAA (**Figure 3A**). In IG, brassinosteroid and nitrogen metabolism peaked at 16 DAA, linoleic and galactose metabolism at 23 DAA, and cysteine and methionine metabolism at 30 DAA (**Figure 3B**). Amino acid metabolism in both SG and IG strongly responded to nitrogen throughout development.

Nitrogen application significantly increased glutamate and asparagine levels across development (**Supplementary Figure S3**). Further analysis revealed elevated glutamine and proline at 16 DAA, declining thereafter, with nitrogen enhancing glutamine content. By 30 DAA, lysine accumulated, further boosted by nitrogen (**Figure 3C**). These shifts underscore nitrogen’s role in modulating amino acid metabolism during grain development.

### 3.4 Transcriptome analysis of superior and inferior grain in responding to nitrogen application

Full-length transcriptome sequencing was performed on the PacBio Sequel system and short-read RNA-Seq on the Illumina HiSeq platform. In total, 36 samples generated 57.48 Gb of data (Q30 ≥ 85%). PacBio sequencing yielded 560,075 circular consensus sequencing (CCS) reads, including 526,768 full-length non-chimeric (FLNC) reads; after clustering and redundancy removal, 124,425 non-redundant transcripts were obtained. Illumina libraries yielded ~19–27 million clean reads each, with Q30 > 90% and GC content of ~53–56%. Detailed sequencing statistics are provided in **Supplementary Tables S3–S4**.

Sample correlation analysis showed clear separation across developmental stages, with samples clustering tightly by time point (**Supplementary Figure S4A**). To explore nitrogen’s impact, DEGs were compared across six conditions for SG and IG. In SG, nitrogen application (CS_vs_NS) drove upregulation of 559, 472, and 650 DEGs at 16, 23, and 30 DAA, respectively, with 438, 586, and 513 downregulated. In IG (CI_vs_NI), upregulated DEGs were 683, 383, and 425, and downregulated ones were 383, 509, and 425 at the same stages (**Supplementary Figure S4B**).

**Figure 4:**
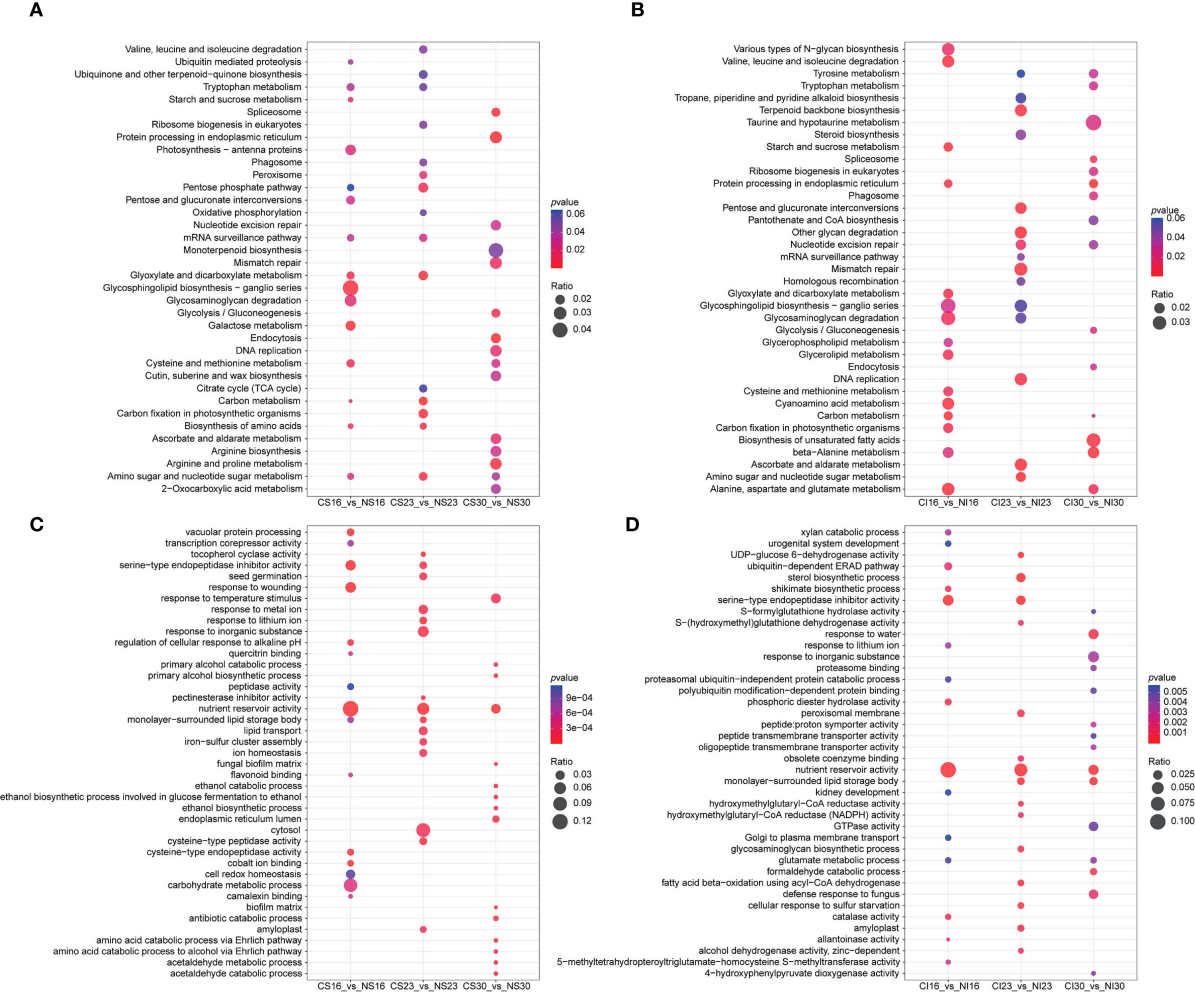
KEGG and GO pathway enrichment analyses. **(A)** KEGG analysis of CS vs. NS; **(B)** KEGG analysis of CI vs. NI; **(C)** GO analysis of CS vs. NS; **(D)** GO analysis of CI vs. NI.

KEGG analysis of SG and IG DEGs revealed dynamic shifts across the top 15 enriched pathways. For SG, early enrichment at 16 DAA featured galactose and amino acid biosynthesis, shifting to carbon metabolism at 23 DAA, and protein processing and glycolysis by 30 DAA (**Figure 4A**). For IG, enriched pathways at 16 DAA included carbon metabolism, glutamate metabolism, and starch metabolism; at 23 DAA, amino sugar metabolism, ascorbate metabolism, and glycan degradation; and at 30 DAA, protein processing, fatty acid biosynthesis, and glutamate metabolism (**Figure 4B**). Shared pathways between SG and IG included carbon metabolism and starch metabolism at 16 DAA, amino sugar metabolism at 23 DAA, and protein processing and spliceosome at 30 DAA.

GO enrichment analysis revealed that nitrogen treatment regulated several key biological processes in both SG and IG at different developmental stages (**Figures 4C, D**). In SG, DEGs were enriched for “vacuolar protein processing” and “serine-type endopeptidase inhibitor activity” at 16 DAA, “pectinesterase inhibitor activity” and “cell wall modification” at 23 DAA, and “antibiotic catabolic process” and “nutrient reservoir activity” at 30 DAA, reflecting enhanced protein synthesis, protease inhibition, cell wall remodeling, and defense-related metabolism. In IG, GO terms such as “serine-type endopeptidase inhibitor activity” were enriched at 16 DAA, “sterol biosynthetic process” at 23 DAA, and “lipid storage” and “fatty acid beta-oxidation” at 30 DAA, indicating that nitrogen promoted not only protein-related processes but also lipid metabolism and energy storage during late grain development. These results suggest both shared (such as protease inhibition) and position-specific (such as lipid metabolism in IG) effects of nitrogen on seed developmental processes.

These findings reveal nitrogen’s dynamic role in seed development, driving a shift from early carbon, starch, and amino acid metabolism (16 DAA) to late protein processing, glycolysis, and energy pathways (30 DAA), with SG and IG exhibiting distinct, stage-specific responses in biosynthesis and catabolism.

**Figure 5:**
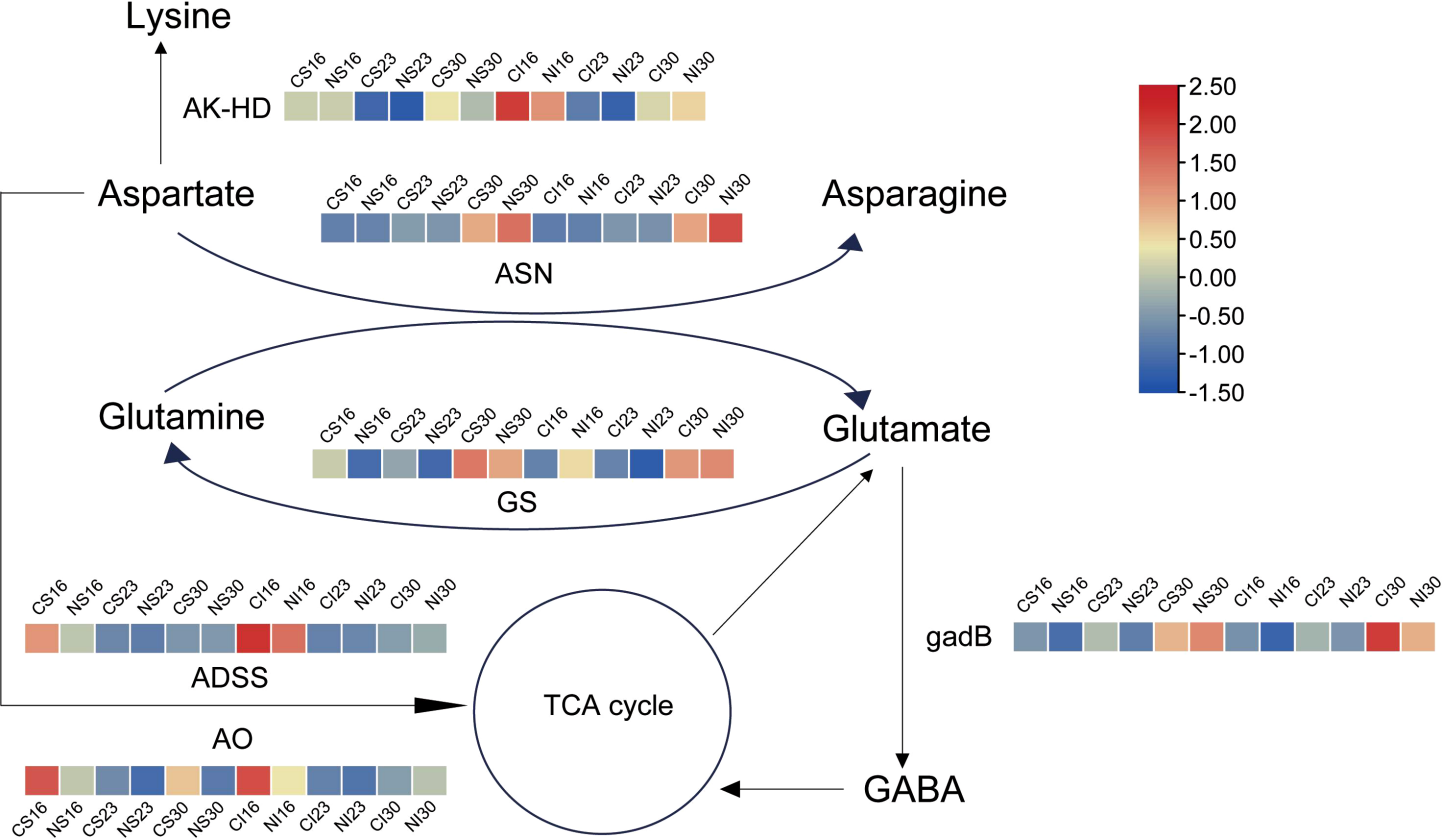
Expression patterns of genes tied to glutamate and asparagine metabolism. *AK-HD*, Bifunctional aspartokinase; *ASN*, Asparagine synthetase; *GS*, Glutamine synthetase; *ADSS*, Adenylosuccinate synthetase; *AO*, Aspartate oxidase; *gadB*, Glutamate decarboxylase.

Based on the transcriptional analyses described above, gene expression patterns in glutamate and aspartate metabolism were mapped via heatmaps (**Figure 5**). During seed development, genes related to aspartate and glutamate metabolism, including asparagine synthetase (*ASN*), bifunctional aspartokinase (*AK-HD*), adenylosuccinate synthetase (*ADSS*), aspartate oxidase (AO), glutamine synthetase (*GS*), and glutamate decarboxylase (*gadB*), displayed dynamic expression changes (**Figure 5**). The expression levels of *ASN* and *GS* were increased at 30 DAA. These patterns are consistent with the late-stage enrichment of protein processing pathways observed in the KEGG analysis, suggesting their possible involvement in nitrogen-mediated seed development. Nitrogen markedly boosted *ASN* in both SG and IG at 30 DAA, consistent with enhanced amino acid metabolism. At 16 DAA, nitrogen downregulated *GS*, *gadB*, *ADSS*, and *AO* in SG—aligning with early amino acid biosynthesis trends—while in IG, it upregulated *GS* and suppressed *AK-HD*, *gadB*, *ADSS*, and *AO*, reflecting glutamate metabolism enrichment. By 30 DAA, nitrogen reduced *AO* in SG and *gadB* in IG, fine-tuning degradation. These patterns highlight nitrogen’s role in amplifying glutamate and asparagine synthesis, complementing the stage-specific metabolic reprogramming.

### 3.5 WGCNA analysis of DEGs

**Figure 6:**
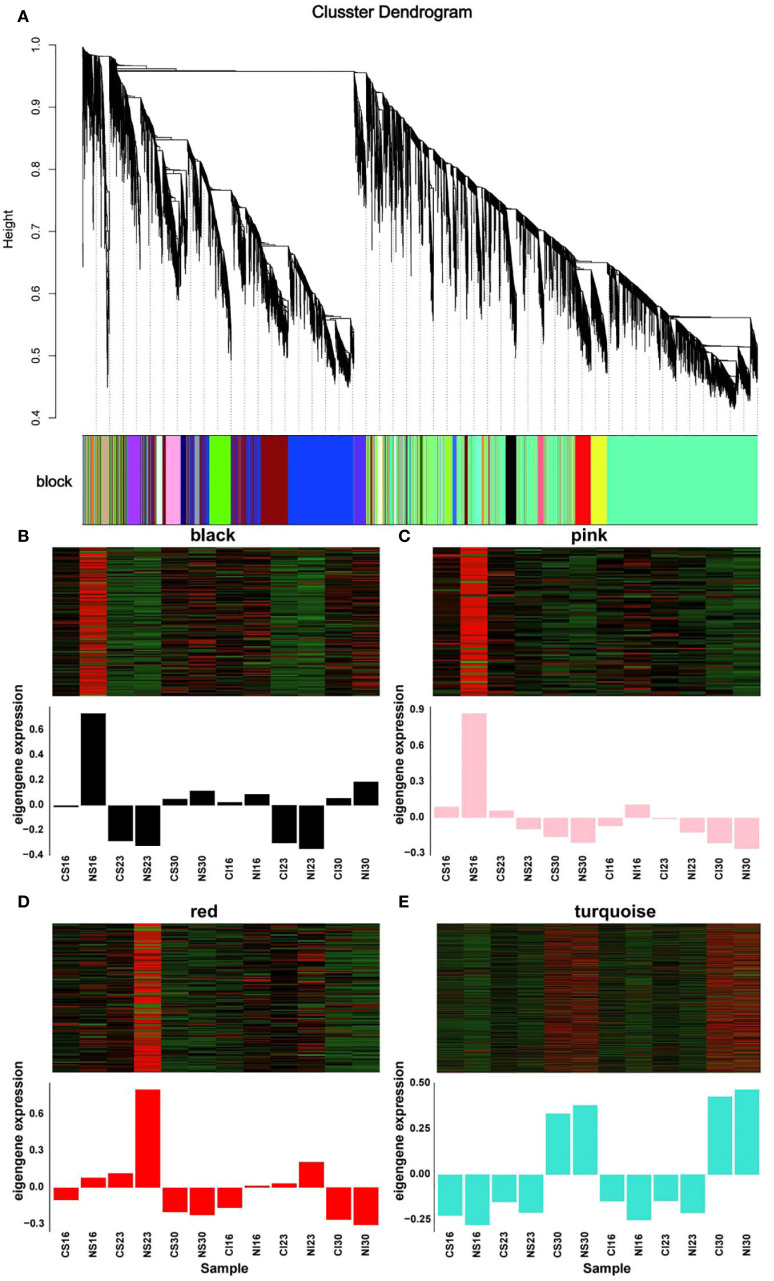
Weighted Gene Co-expression Network Analysis (WGCNA) of differentially expressed genes. **(A)** Gene clustering dendrogram. Each color in the block below the dendrogram represents a distinct co-expression module. **(B–E)** Heatmaps (top) and eigengene expression profiles (bottom) for the four key nitrogen-responsive modules: **(B)** black, **(C)** pink, **(D)** red, and **(E)** turquoise.

To further dissect the metabolic shifts observed, WGCNA was applied to 36,929 DEGs. Genes with *FPKM* < 0.1 across all samples or median absolute deviation (MAD) > 1 were excluded, leaving 18,853 genes for analysis. Clustering of the 12 sample groups was performed according to developmental stages (16, 23 and 30 DAA). At 16 and 23 DAA, samples separated by grain type (superior vs. inferior), while at 30 DAA nitrogen treatment drove clustering (**Supplementary Figure S5**). Using *FPKM* values, *DEGs* with high correlation were grouped into 11 distinct modules, each assigned a colour, with the turquoise module being the largest (**Supplementary Table S5**, **Figure 6A**). Focusing on the nitrogen-responsive modules - black, pink, red and turquoise - the expression patterns revealed stage-specific responses. At 16 DAA, black and pink module genes were up-regulated by nitrogen in both cereal types. Red module genes showed nitrogen-induced upregulation at 16 and 23 DAA, while turquoise module genes peaked at 30 DAA under nitrogen treatment (**Figures 6B–E**). These trends are consistent with the role of nitrogen in enhancing amino acid metabolism, particularly glutamate and asparagine synthesis, as seen in previous heat map analyses (**Figure 5**).

**Figure 7:**
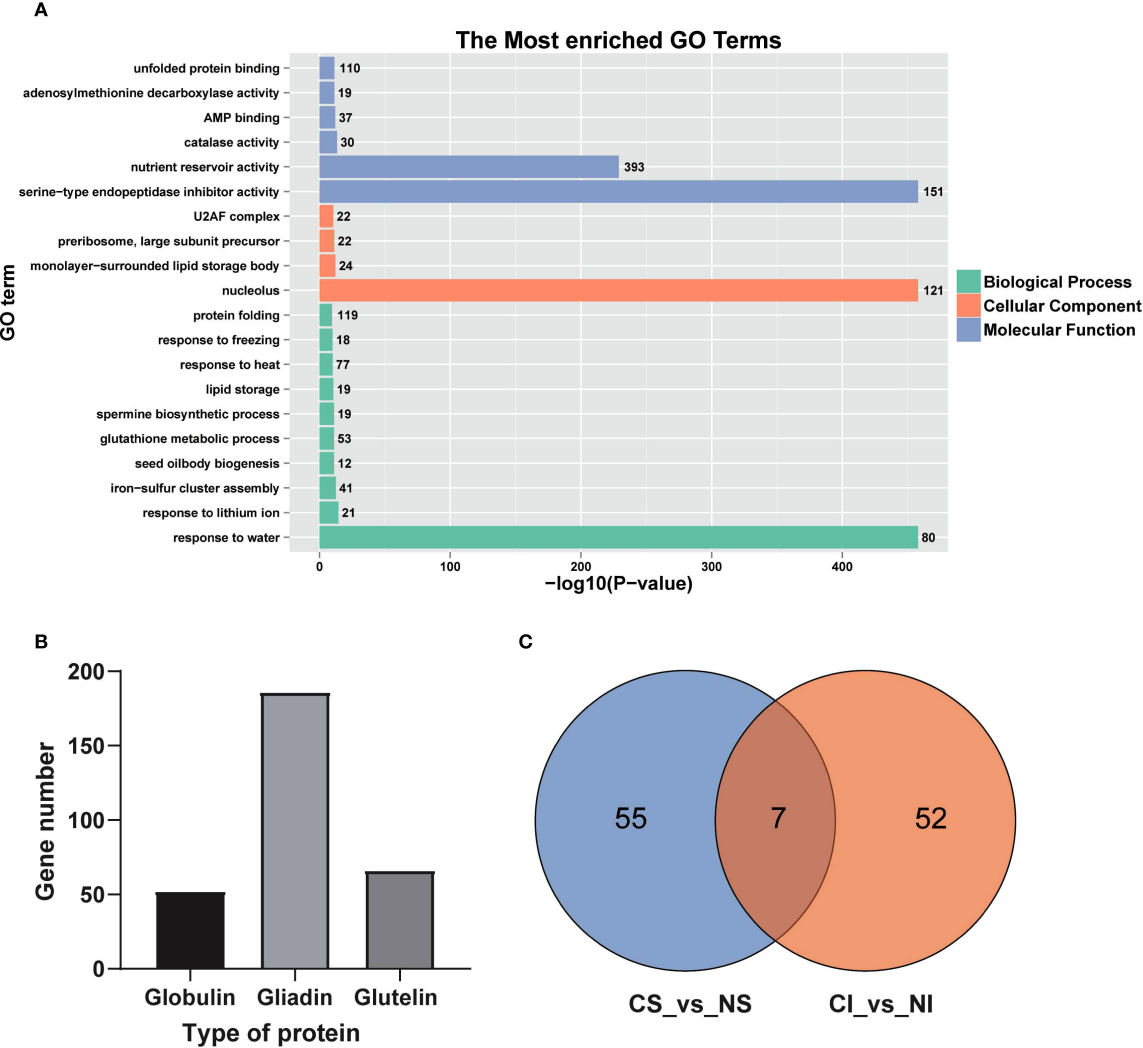
GO pathway enrichment and DGE analysis of nutrient reservoir activity. **(A)** GO enrichment of genes in black, pink, red, and turquoise module; **(B)** Gene counts by protein type; **(C)** Venn diagram of DGEs in SG and IG under nitrogen treatment.

To further characterize the nitrogen-responsive modules, KEGG and GO enrichment analyses were conducted on genes within the black, pink, red, and turquoise modules. KEGG results revealed enrichment in metabolic pathways critical for protein synthesis, including endoplasmic reticulum protein processing and amino acid metabolism (e.g., lysine, valine, leucine, isoleucine, cysteine, methionine, tryptophan, β-alanine, histidine, arginine, proline, alanine, aspartate, glutamate, and phenylalanine metabolism) (**Supplementary Figure S6**). GO analysis highlighted enrichment in nucleolus, response to water, serine-type endopeptidase inhibitor activity, and nutrient reservoir activity (**Figure 7A**). Nutrient reservoir activity, pivotal for storage substance accumulation during seed filling, involved 393 genes, predominantly encoding gliadins (notably α-gliadins), globulins, and glutelins (**Figure 7B**). Notably, superior and inferior grains exhibited distinct responses to nitrogen among these DEGs, sharing only seven common DEGs (**Figure 7C**). These findings highlight the differences in nitrogen responses between superior and inferior grains, providing a basis for optimizing nitrogen fertilizer strategies. The goal of this optimization is to narrow the developmental disparities between grain types and ultimately increase total yield.

### 3.6 Effects of overexpression BiGli1 and BiGli2 genes on seed vigor

Transcript abundance in transgenic Arabidopsis OE lines was determined by RT-qPCR. All selected OE lines showed high expression of the target genes (**Supplementary Figure S7**), confirming successful overexpression. Protein accumulation in these lines was further verified by Western blot analysis using an anti-GFP antibody (**Supplementary Figure S8**). These results demonstrate that *BiGli1* and *BiGli2* were robustly expressed at both the transcript and protein levels in the selected transgenic lines.

Nitrogen application was found to influence amino acid metabolism and protein biosynthesis in superior and inferior grains, with effects observed at physiological, transcriptional, and metabolic levels. Gene expression profiling revealed distinct patterns between superior and inferior grains under nitrogen treatment. From these findings, two α-gliadin-related genes, *BiGli1* and *BiGli2*, which exhibit responsiveness to nitrogen in inferior grains, were identified (**Supplementary Table S6**). Their roles were validated through overexpression in *Arabidopsis thaliana*, with seed phenotypes and germination traits evaluated under aging conditions. Stereomicroscopic imaging (scale bar: 1000 μm) revealed that in *BiGli1*-overexpressing lines, OE2 had a significantly greater seed surface area and length than the wild-type Columbia (Col) (*P* < 0.01), while OE4 showed a significantly larger surface area (*P* < 0.05). In BiGli2-overexpressing lines, OE1 exhibited significant increases in seed surface area, length, and width (*P* < 0.05); OE3 displayed significantly greater surface area and length (*P* < 0.05); and OE6 showed significant increases in all three traits (*P* < 0.05). Among the *BiGli2* lines, OE6 demonstrated the most pronounced improvements, with seed surface area and length increased by 18.4% and 12.7%, respectively, relative to Col (**Table 2**).

**Table 2:** Seed length, width and surface area of Arabidopsis plants overexpressing *BiGli1* and *BiGli2* genes.

Indices	Col	*BiGli1*			*BiGli2*		
		OE2	OE4	OE6	OE1	OE3	OE6
Surface area(mm^2^)	0.087	0.097**	0.092*	0.091	0.094**	0.093**	0.103**
Length(mm)	0.426	0.451**	0.440	0.436	0.453**	0.454**	0.480**
Width(mm)	0.260	0.269	0.263	0.264	0.274*	0.260	0.274*

* indicates significant difference compared with the control. * means significant difference at 0.05 level, ** means significant difference at 0.01 level.

**Figure 8:**
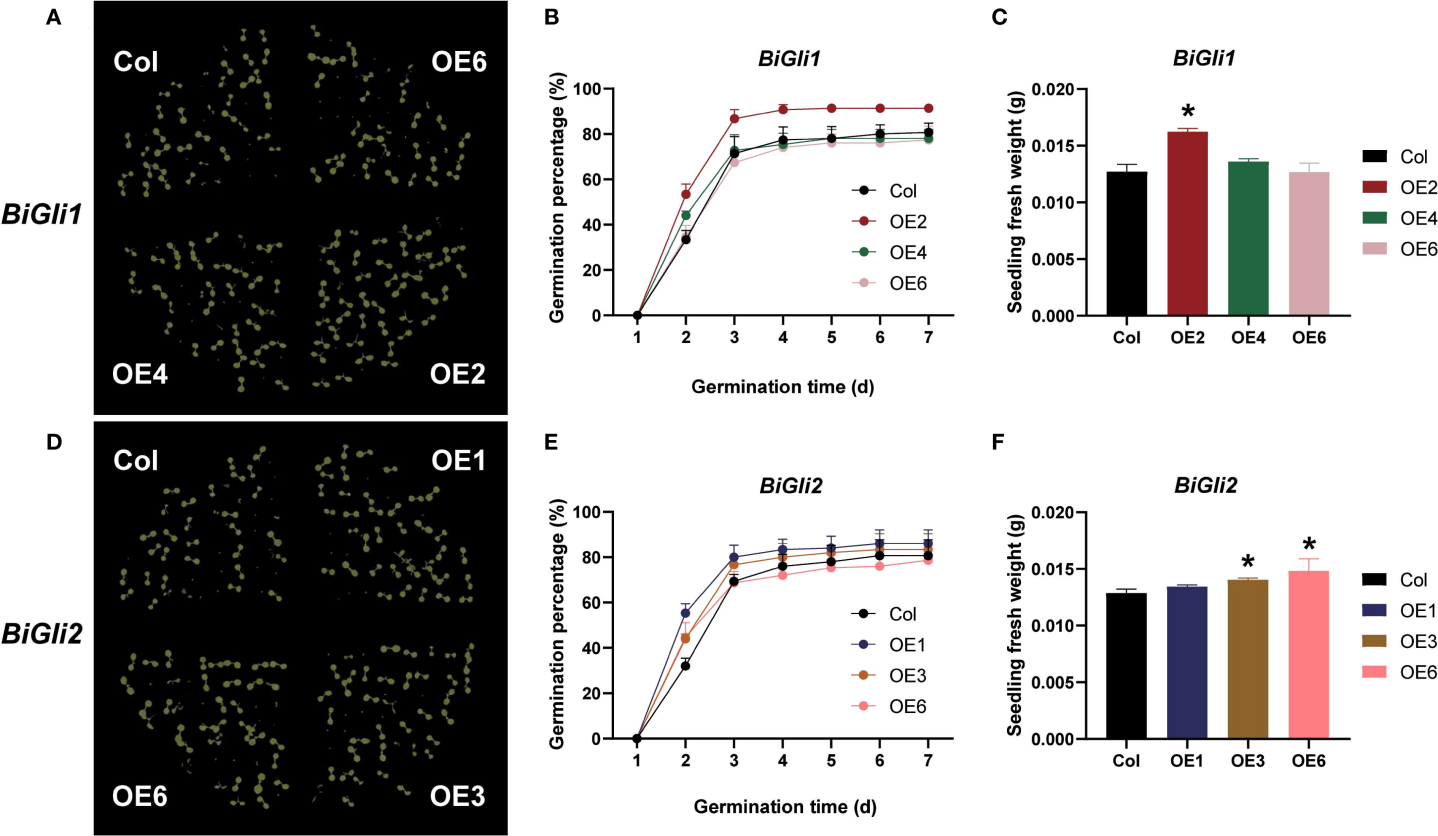
Effects of aging treatment on seed germination in Arabidopsis plants overexpressing *BiGli1* and *BiGli2* genes. **(A, D)** Images of seed germination on day 7 for aged Arabidopsis seeds overexpressing *BiGli1* **(A)** and *BiGli2* **(D)**. **(B, E)** Germination percentages of aged Arabidopsis seeds overexpressing *BiGli1* **(B)** and *BiGli2* **(E)**. **(C, F)** Seedling fresh weights of aged Arabidopsis seeds overexpressing *BiGli1* **(C)** and *BiGli2* **(F)**. An asterisk (*) denotes a significant difference compared to the wild-type Columbia (Col) at the *P*<0.05 level.

**Figure 9:**
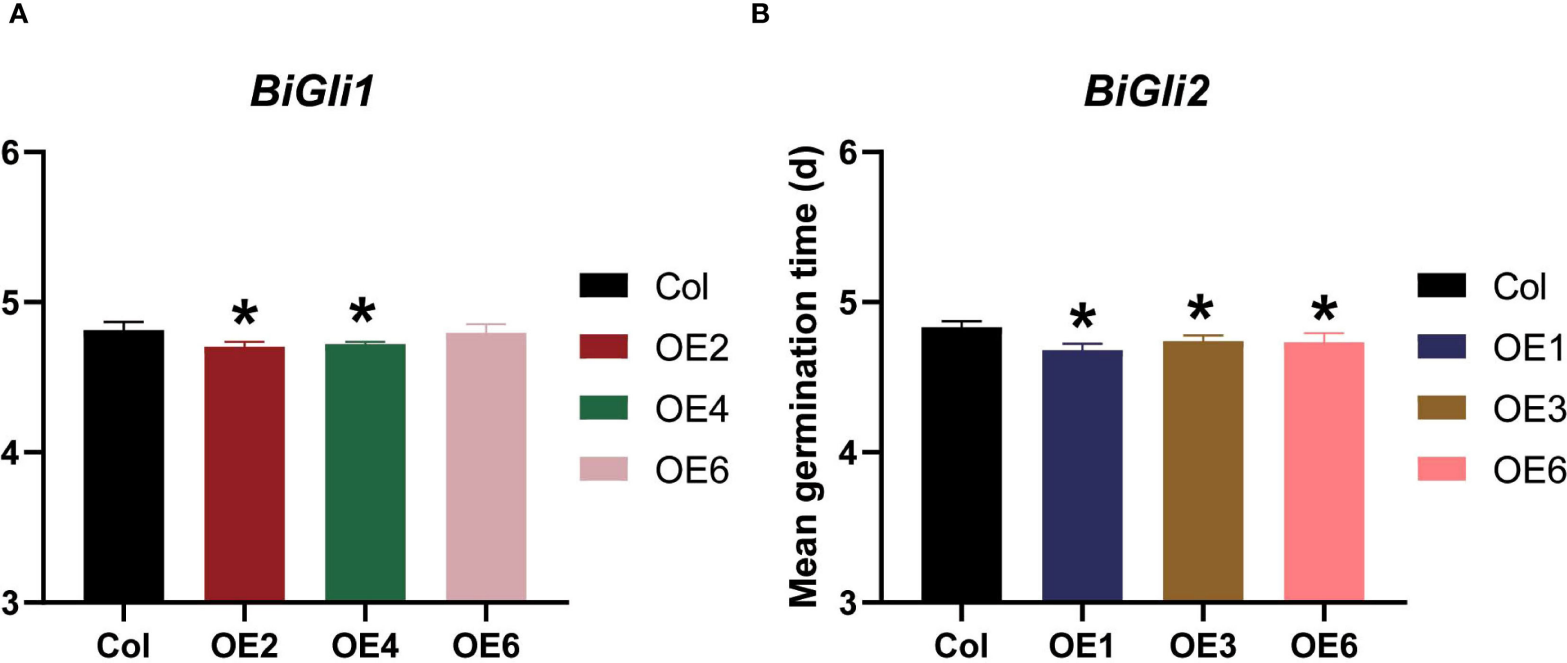
Effects of aging treatment on mean germination time of Arabidopsis seeds overexpressing *BiGli1* **(A)** and *BiGli2* **(B)** genes. An asterisk (*) indicates a significant difference compared to the wild-type Columbia (Col) at the *P*<0.05 level.

After aging, the germination phenotypes (**Figures 8A, D**) were observed, and the germination percentage and seedling fresh weight were assessed. The *BiGli1*-overexpressing OE2 line exhibited significantly higher germination percentage and seedling fresh weight (*P* < 0.05) compared to Col, with a 26.9% increase in fresh weight (**Supplementary Table S7**). Both OE2 and OE4 lines outperformed Col in germinated seed count on day two (**Figures 8B, C**) and displayed significantly shorter mean germination times (*P* < 0.05) (**Figure 9A**). For *BiGli2*-overexpressing lines, germination percentage did not differ significantly from Col (*P*>0.05), but OE1 and OE3 lines had higher germinated seed counts on day two (**Figure 8E**), while OE3 and OE6 lines showed significantly greater seedling fresh weight (*P*<0.05) (**Figure 8F**). Additionally, all three *BiGli2*-overexpressing lines (OE1, OE3, OE6) exhibited significantly shorter mean germination times (*P*<0.05) than Col (**Figure 9B**). These findings suggest that *BiGli1* and *BiGli2* enhance seed vigor by modulating seed size and germination performance, notably through a unique responsiveness observed in inferior grains of *Bromus inermis*, offering preliminary insights into improving grain quality, particularly in inferior grains, under nitrogen regulation.

## 4 Discussion

### 4.1 Nitrogen application regulates nitrogen and amino acid metabolism in superior and inferior grain development

Nitrogen metabolism underpins seed development, with nitrate and ammonium serving as primary inorganic nitrogen sources. Roots absorb nitrate, converting it to ammonium, which is then assimilated into organic nitrogen via the glutamine synthetase (*GS*)/glutamate synthase (*GOGAT*) cycle (Liu et al., 2022). This organic nitrogen, transported to stems and leaves, forms free amino acids (e.g., aspartate, asparagine, glutamate, glutamine) that are later mobilized to seeds during reproductive growth to synthesize storage proteins like gliadin and glutelin. Glutamate and aspartate, critical in the *GS/GOGAT* pathway and nitrogen transport, constitute over 20% of total seed protein (Masclaux-Daubresse et al., 2010). Here, at 16 DAA, nitrogen application suppressed the expression of aspartate degradation genes (*AK-HD*, *ADSS*, *ASP*) more markedly in superior grains than in inferior ones, favoring aspartate accumulation. Notably, *GS* expression was downregulated in superior grains but upregulated in inferior grains at 16 DAA, suggesting nitrogen enhances assimilation and transport more effectively in inferior grains. Prior studies corroborate that *GS* overexpression boosts ammonia assimilation and nitrogen remobilization (Zeng et al., 2017; Hu et al., 2018). Additionally, asparagine, a key nitrogen transport compound in cereals (Miflin and Habash, 2002), increased significantly in seeds with nitrogen application (Wilson et al., 2020), with *ASN* expression markedly upregulated at 30 DAA in both grain types, further amplified by nitrogen.

Amino acid metabolism, vital for protein synthesis, responded strongly to nitrogen. In rice, nitrogen elevates free and protein-bound amino acid levels (Zhao et al., 2015), with inferior grains showing heightened sensitivity (Zhang et al., 2017). Similarly, in smooth bromegrass, nitrogen boosted glutamine early in seed development, aiding nitrogen transport, and elevated asparagine and glutamate levels, catalyzed by asparagine synthetase (*THP9*). Previous maize studies link *THP9* overexpression to increased protein accumulation (Huang et al., 2022), a trend mirrored here, where nitrogen enhanced protein content in both SG and IG.

Nitrogen fertilization enhanced storage protein accumulation in both SG and IG, with a proportionally greater benefit observed in IG. A plausible explanation is that adequate nitrogen availability alleviates the inherent resource competition between grain types by increasing the overall pool of assimilates and potentially modifying source-sink dynamics (Abdelrahman et al., 2020). While this study offers valuable insights into the responses of SG and IG to nitrogen application, several limitations should be noted. The experiments were conducted only in the field, introducing environmental variability, and included just two nitrogen levels, precluding a full dose–response assessment.

### 4.2 Nitrogen application regulates protein synthesis in superior and inferior grains

Seed storage proteins, accumulated during development, determine yield and quality. Environmental factors, particularly nitrogen, exert a stronger influence on protein content than genetics (Wan et al., 2012). In wheat, nitrogen doubles protein levels (Godfrey et al., 2010), with moderate application optimizing both yield and protein (Zhang et al., 2016). This study found nitrogen significantly increased gliadin and glutelin content in smooth bromegrass seeds, enhancing seed vigor and slightly reducing germination time, consistent with our preprint findings (Ou et al., 2025). KEGG and GO analyses of nitrogen-responsive genes (via WGCNA) revealed enriched pathways in protein synthesis (e.g., endoplasmic reticulum processing, amino acid metabolism) and processes like nutrient reservoir activity and protein folding (Barneix, 2007). Yu et al (Yu et al., 2017). and Chope et al (Chope et al., 2014). reported similar increases in wheat gliadin and glutelin under high nitrogen, with differential gene responses (e.g., α-gliadin dominance) between superior and inferior grains here suggesting tailored regulation. High nitrogen also elevated total gliadin (Zhen et al., 2020), underscoring its role in storage protein modulation. Future research should target inferior grain protein synthesis pathways to optimize smooth bromegrass seed quality.

### 4.3 Overexpression of gliadin genes BiGli1 and BiGli2 enhances seed viability

Gliadins, comprising 40–50% of grain protein in Poaceae (Li et al., 2018), are classified by electrophoresis into α/β- (55%), γ- (30%), and ω-gliadins (15%) (Qi et al., 2006; Wujun and Yu, 2019), influencing flour properties and celiac disease (Ting et al., 2020). While silencing efforts reduce allergenicity (Sánchez-León et al., 2021; Marín-Sanz and Barro, 2022), smooth bromegrass lacks such concerns. In this study, the overexpression of *BiGli1* and *BiGli2* (annotated as α-gliadins) in Arabidopsis increased seed size (surface area, length, and width) compared to Col controls (Section 3.6, **Figure 8F**), which indicates that these genes promote seed development. Storage proteins fuel germination, as seen in rice where *PcG-OsFIE1* and *Snrk1α1/α2* regulate protein and amino acid availability (Huang et al., 2016; Henninger et al., 2021). Higher globulin correlates with better germination (Peng et al., 2022). Under aging, *BiGli1* and *BiGli2* overexpressing seeds exhibited shorter germination times and greater seedling weight (**Figures 9A, B**), suggesting improved vigor and stress resistance. However, the mechanisms driving these effects require further study.

Our results highlight the central role of *BiGli1* and *BiGli2* in enhancing seed vigour through nitrogen-mediated regulation in smooth bromegrass. Compared to other Poaceae species, such as wheat, where gliadins primarily influence flour quality (Zhen et al., 2020), smooth bromegrass uses *BiGli1* and *BiGli2* to enhance germination and stress resistance, highlighting a species-specific adaptation. In practical terms, these findings suggest a dual strategy of nitrogen optimisation and genetic modification as a promising way to improve seed quality in forage grasses under variable environmental conditions. To fully elucidate their mechanisms, future work should investigate the temporal dynamics of *BiGli1* and *BiGli2* expression and their downstream effects on protein-amino acid metabolism under nitrogen gradients, possibly using proteomics for deeper resolution. Future research could leverage advanced breeding and molecular tools such as CRISPR/Cas9-mediated genome editing to modify key genes regulating storage protein synthesis and nutrient allocation. While our study reveals important molecular features underlying nitrogen-mediated regulation of seed vigor in smooth bromegrass, it was conducted on a single cultivar. As genotype- and grain position–dependent differences in responses to nitrogen may exist, multi-omics analyses across diverse cultivars are needed to validate and extend these findings. Such comparative work will help determine the broader applicability of the mechanisms identified here and provide a stronger basis for breeding and management strategies.

## 5 Conclusion

**Figure 10:**
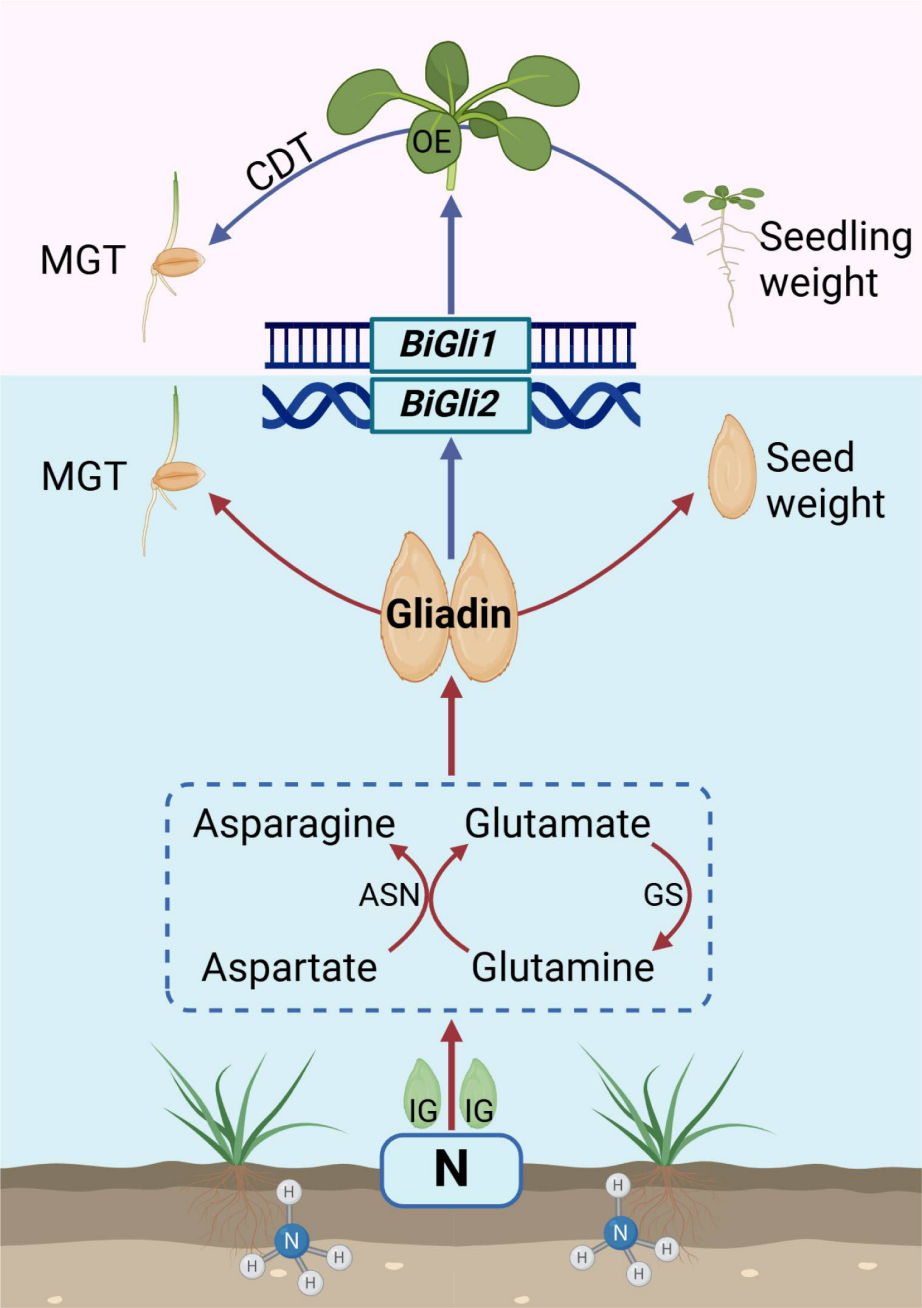
Model description of nitrogen application enhancing seed vigor (Created in https://BioRender.com).

Nitrogen application significantly increased both the dry and fresh weights of superior and inferior smooth bromegrass grains, primarily by elevating seed protein content through enhanced gliadin and glutelin accumulation. At the metabolic and transcriptional levels, nitrogen markedly influenced amino acid metabolism during seed development, upregulating glutamate and asparagine levels in both grain types to facilitate nitrogen transport and protein synthesis. Overexpression of *BiGli1* and *BiGli2* in Arabidopsis enhanced seed vigor, supporting the observed positive effects of nitrogen application on smooth bromegrass seeds and modelling our hypothesis model (**Figure 10**). This was evidenced by increased seed size and seedling fresh weight, alongside reduced mean germination time. Collectively, these findings demonstrate that nitrogen application, coupled with *BiGli1* and *BiGli2* activity, offers a robust strategy to improve seed quality and vigor in smooth bromegrass.

## Data Availability

The raw transcriptome sequencing data generated in this study have been deposited in the National Genomics Data Center (NGDC, https://ngdc.cncb.ac.cn; accessed on 27 January 2025) under BioProject PRJCA035644, and in the NCBI Sequence Read Archive (SRA; https://www.ncbi.nlm.nih.gov/sra; accession date: 8 August 2025) under BioProject accession number PRJNA1304003. The raw metabolome data have also been deposited in NGDC under BioProject PRJCA035655 (accessed on 28 January 2025).
