# Supplementary material for: Integrated multi-omics analysis reveals the role of nitrogen application in seed storage protein metabolism and improvement of inferior grains in smooth bromegrass
Source: Front Plant Sci. 2025 Sep 12;16:1605073. doi: 10.3389/fpls.2025.1605073 (PMC12463976; doi:10.3389/fpls.2025.1605073)
Supplement: Supplementary file 1 [file Supplementaryfile1.docx]

Integrated multi-omics analysis reveals the role of nitrogen application in seed storage protein metabolism and improvement of inferior grains in smooth bromegrass

Chengming Ou^#a^, Shiqiang Zhao^#a^, Zhicheng Jia^a^, Shoujiang Sun^a^, Juan Wang^a^, Chunjiao Mi^a^, Jinyu Shi^a^, Changran Li^a^, Peisheng Mao^a^*

^a^ College of Grassland Science and Technology, China Agricultural University, Key Laboratory of Pratacultural Science, Beijing Municipality, Beijing 100193, China

*Corresponding author:

Email: [maops@cau.edu.cn](mailto:maops@cau.edu.cn)

Tel: +010-62733311

^#^These authors share first authorship

| 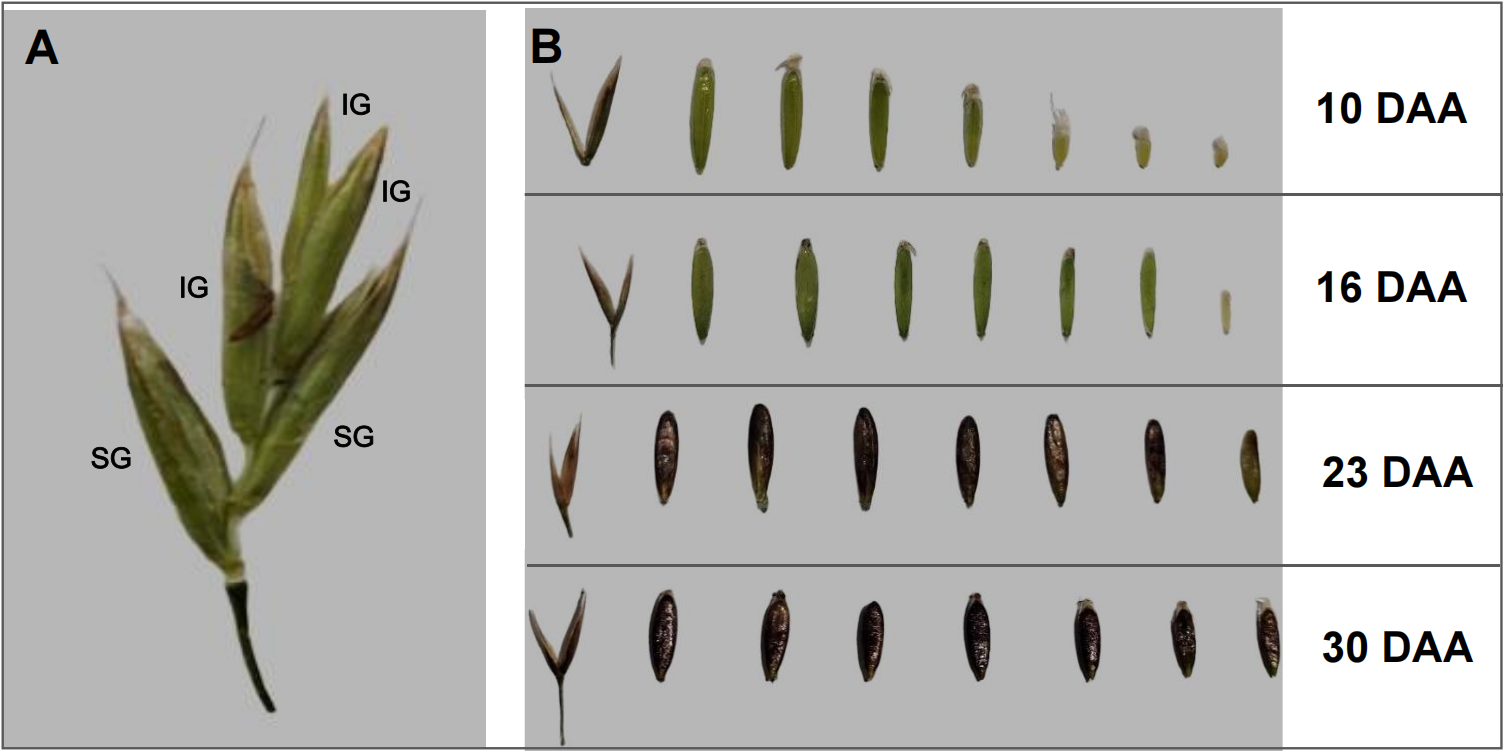 |
| --- |
| Fig. S1 Field presentation of sampling materials and morphological characteristics of smooth bromegrass grains at different developmental stages. (A) Sampling schematic of smooth bromegrass grains. (B) Grains arranged from basal (superior) position on the left to apical (inferior) position on the right within a spikelet at 10, 16, 23, and 30 DAA. |

| 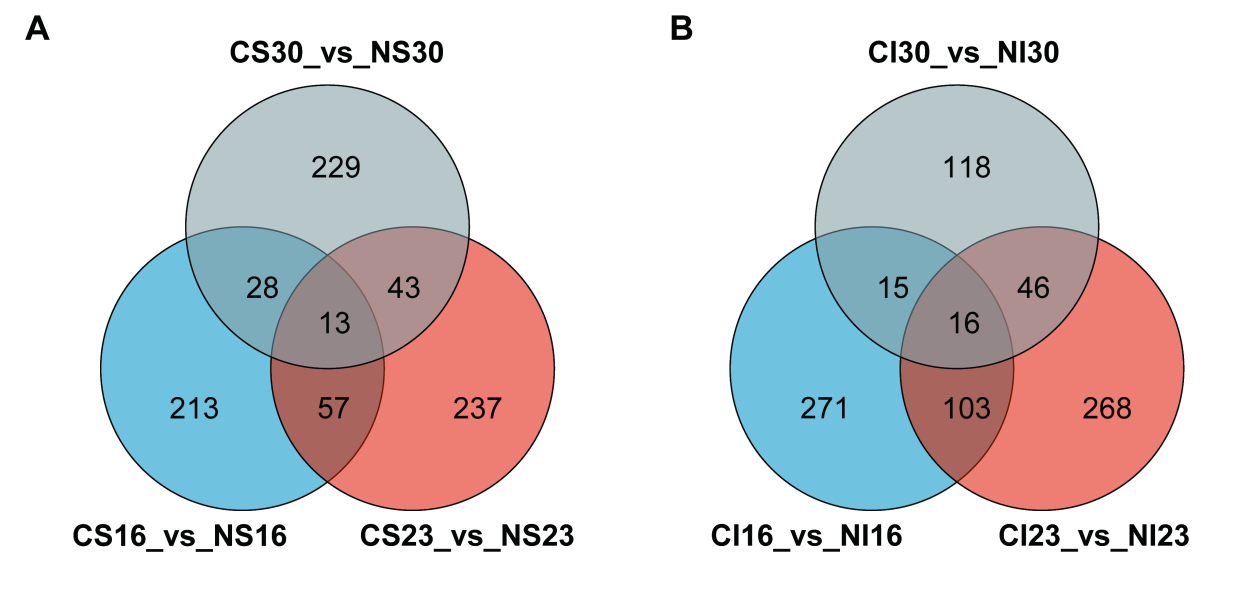 |
| --- |
| Fig. S2 Venn diagram of different metabolites of SG and IG in response to nitrogen. A: SG; B: IG. |

| 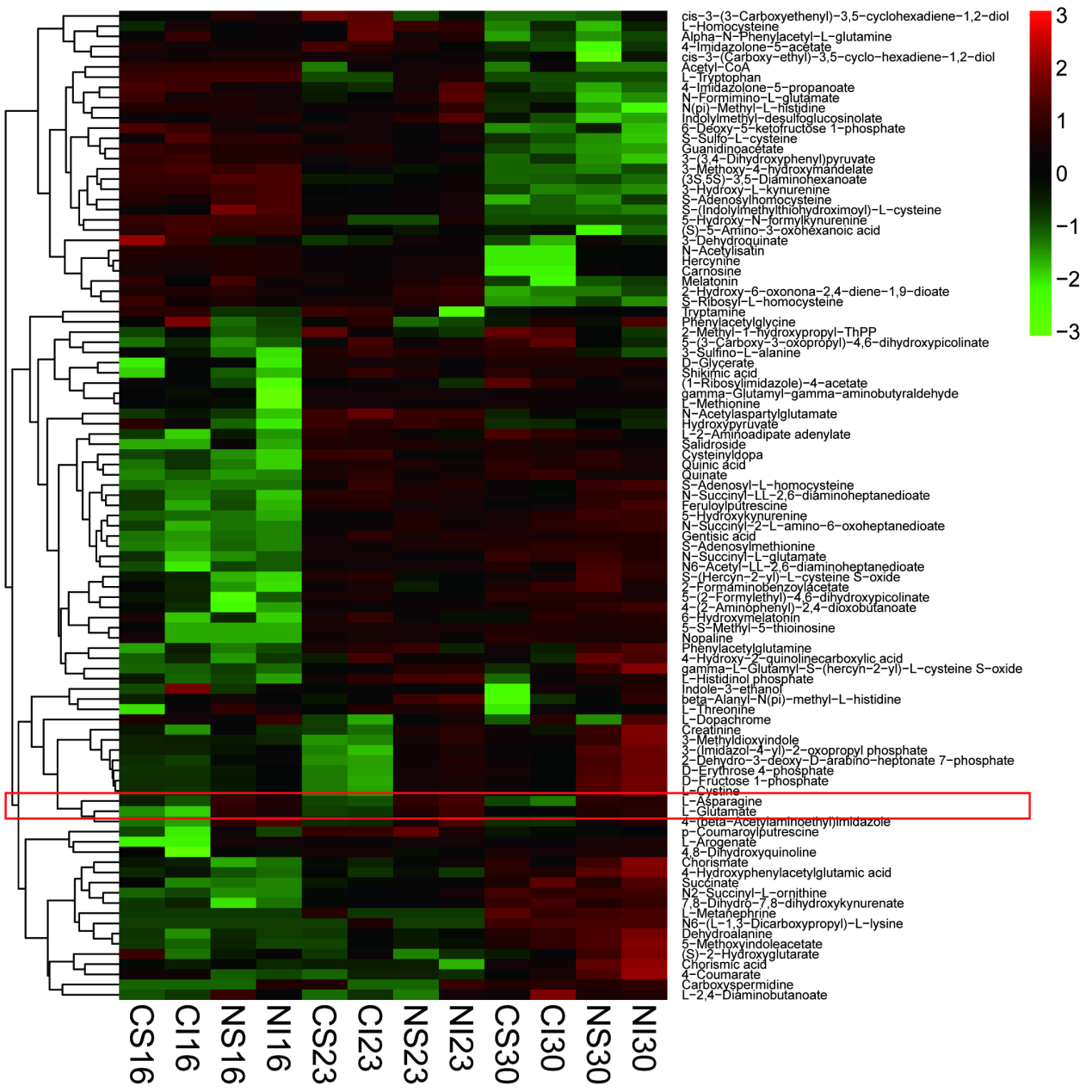 |
| --- |
| Fig. S3 Differential metabolite expression patterns in amino acid metabolism |

| 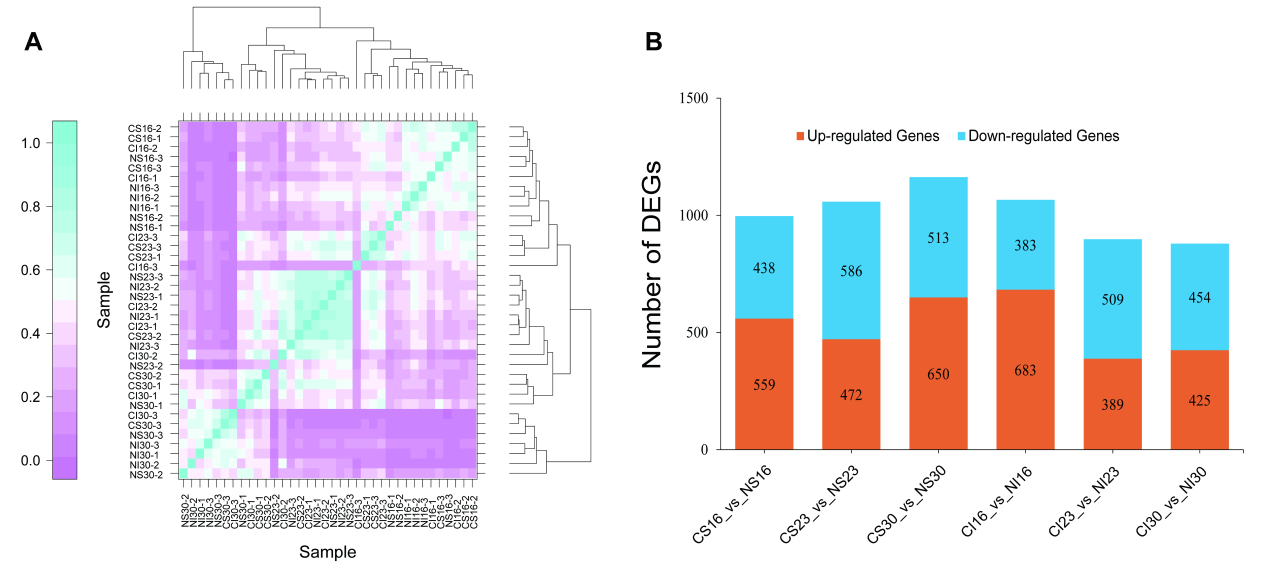 |
| --- |
| Fig. S4 Transcriptome sample status in smooth bromegrass. A: Correlation analysis sample c; B: Differentially expressed genes among different samples. |

| 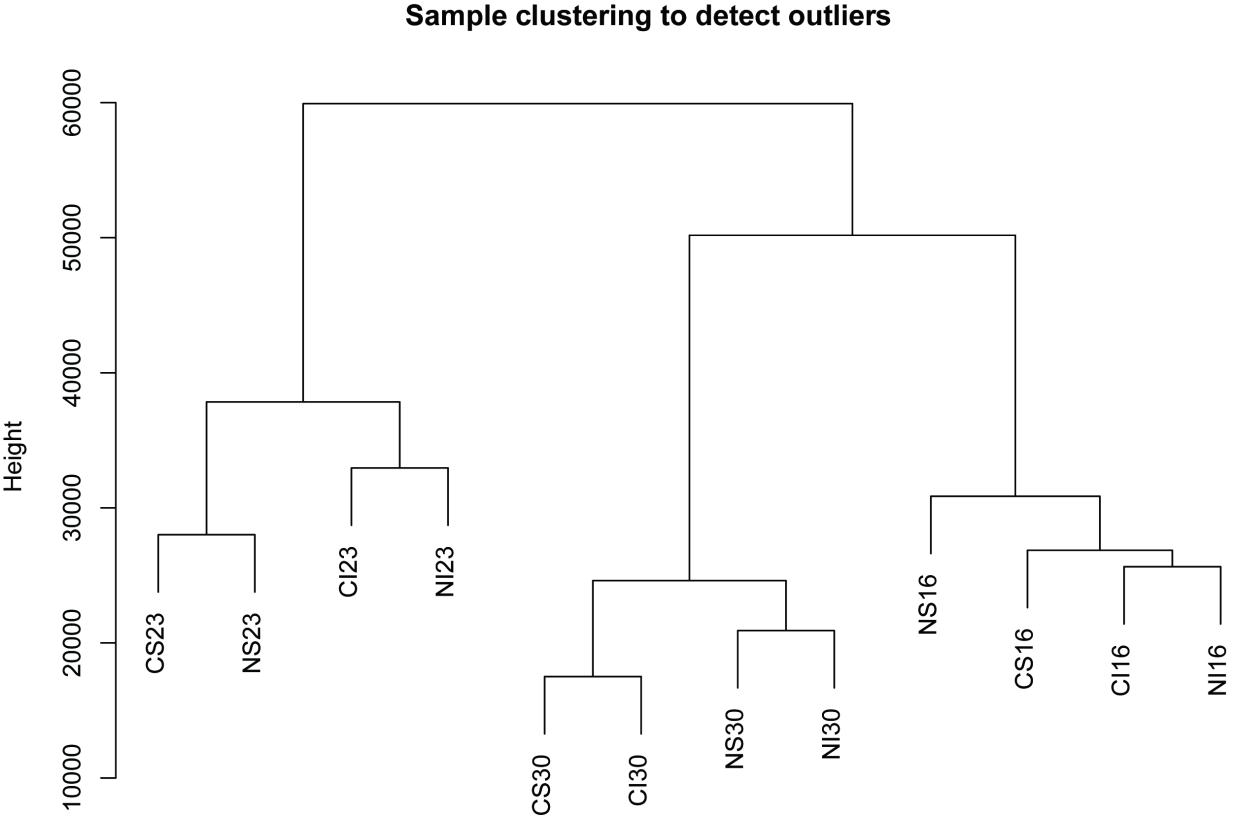 |
| --- |
| Fig. S5 Dendrogram between different samples |

| 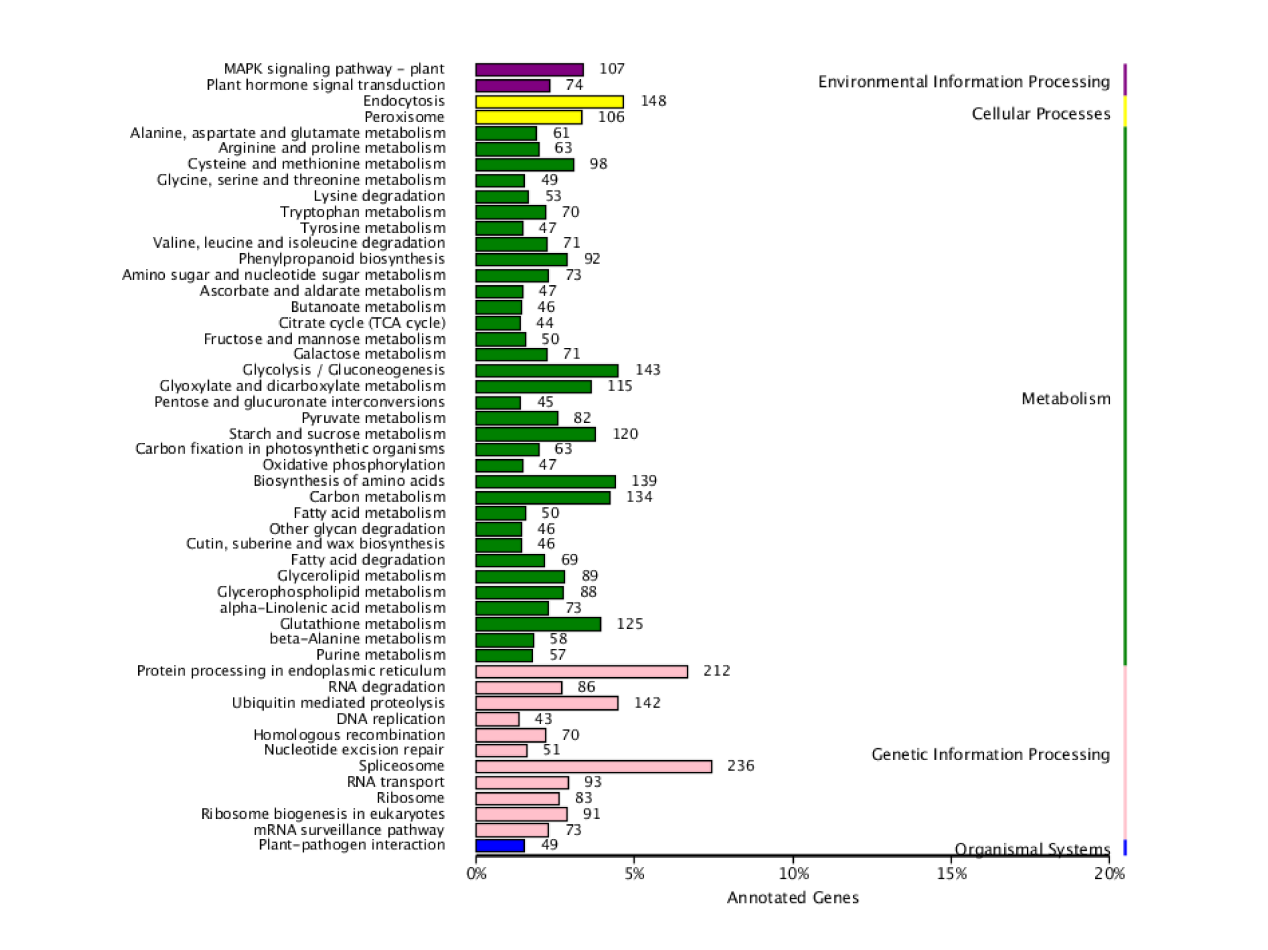 |
| --- |
| Fig. S6 KEGG pathway enrichment analysis of genes in black, pink, red and turquoise module |

| 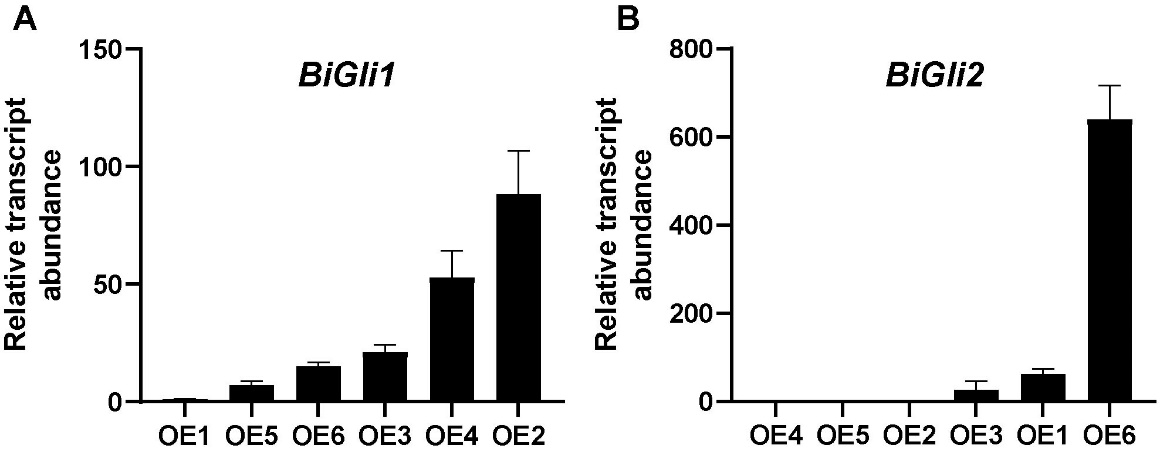 |
| --- |
| Fig. S7 Expression analysis of *BiGli1* and *BiGli2* in transgenic Arabidopsis lines. |

| 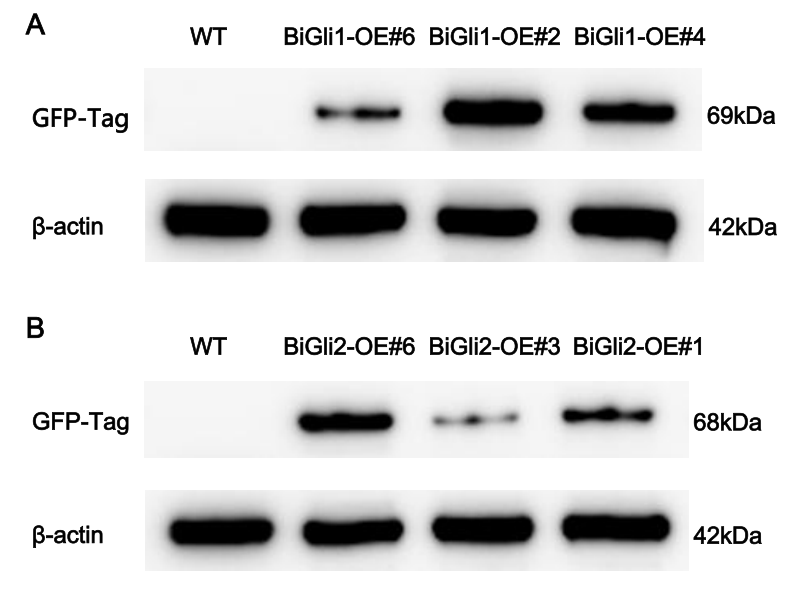 |
| --- |
| Fig. S8 Western blot analysis of BiGli1-GFP and BiGli2-GFP fusion proteins in transgenic Arabidopsis lines. |

Table S1 Primer information

| Primer name | Primer sequence (5’-3’) |
| --- | --- |
| gcBiGli1-F | ggatcttccagagatATGAAGACCTTCCTCGTCCTTG |
| gcBiGli1-R | ctgccgttcgacgatTCACTCGATGTTGGTAGCGGT |
| gcBiGli2-F | ggatcttccagagatATGAAGACCTTCCTCGTCCTTG |
| gcBiGli2-R | ctgccgttcgacgatTTACCACCTCAAAATCAGCAAGTT |
| eBiGli1-F | tgcaggaaagacggttctagaATGAAGACCTTCCTCGTCCTTG |
| eBiGli1-R | gcccttgctcaccattctagaCTCGATGTTGGTAGCGGTGTC |
| eBiGli2-F | tgcaggaaagacggttctagaATGAAGACCTTCCTCGTCCTTG |
| eBiGli2-R | gcccttgctcaccattctagaCCACCTCAAAATCAGCAAGTTG |
| qBiGli1-F | TAGCGTTGCAGACCTTACCC |
| qBiGli1-R | CTCGATGTTGGTAGCGGTGT |
| qBiGli2-F | GCACCAACAACAACAGGTGG |
| qBiGli2-R | GGTACTGCGGTTGAGGTTGT |
| M13-F | GTAAAACGACGGCCAGT |
| M13-R | CAGGAAACAGCTATGAC |
| CX-F | AGAACTCGCCGTAAAGACTGG |
| CX-R | GCCGTAGGTCAGGGTGGTC |
| UBQ10-F | ATTACCCGATGGGCAAGTCA |
| UBQ10-R | CACAAACGAGGGCTGGAACA |

Table S2 Percentage Increase in Dry Weight, Fresh Weight, and Protein Content (N vs. CK)

| Year | DAA | Grain Type | Dry Weight Increase (N vs. CK, %) | Fresh Weight Increase (N vs. CK, %) | Protein Content Increase (N vs. CK, %) |
| --- | --- | --- | --- | --- | --- |
| 2021 | 10 | IG | 22.1 | 7.2 | 35.8 |
| 2021 | 10 | SG | 5.7 | 8.2 | 41.5 |
| 2021 | 16 | IG | 10.2 | 9 | 33.4 |
| 2021 | 16 | SG | 10.2 | 8.3 | 35.2 |
| 2021 | 23 | IG | 7.7 | 15.1 | 22.9 |
| 2021 | 23 | SG | 8.6 | 11.3 | 17.3 |
| 2021 | 30 | IG | 12.2 | 15.3 | 24.4 |
| 2021 | 30 | SG | 8.8 | 9.5 | 24.2 |
| 2022 | 10 | IG | 13.6 | 11.1 | 46.9 |
| 2022 | 10 | SG | 13 | 10.3 | 60.2 |
| 2022 | 16 | IG | 8.8 | 9.5 | 33.2 |
| 2022 | 16 | SG | 5.8 | 7.1 | 32.2 |
| 2022 | 23 | IG | 9 | 7 | 25.3 |
| 2022 | 23 | SG | 7 | 7.7 | 27.1 |
| 2022 | 30 | IG | 11.5 | 25.5 | 31.9 |
| 2022 | 30 | SG | 8.5 | 20.6 | 31 |

Table S3 Sample Illumina HiSeq RNA-Seq data evaluation table

| Sample ID | Raw Reads | Clean Bases | GC Content (%) | Q30 (% |
| --- | --- | --- | --- | --- |
| CS16-1 | 24,582,641 | 7,317,089,296 | 53.38 | 92.13 |
| CS16-2 | 22,557,198 | 6,716,130,372 | 53.26 | 91.52 |
| CS16-3 | 25,679,352 | 7,638,221,866 | 53.35 | 91.8 |
| NS16-1 | 27,245,649 | 8,097,744,088 | 53.03 | 90.75 |
| NS16-2 | 24,208,727 | 7,191,175,988 | 53.28 | 92.18 |
| NS16-3 | 25,867,254 | 7,696,402,230 | 53.57 | 92.42 |
| CI16-1 | 27,558,431 | 8,185,320,542 | 53.46 | 92.44 |
| CI16-2 | 20,449,790 | 6,107,632,756 | 53.78 | 93.51 |
| CI16-3 | 26,354,261 | 7,869,809,444 | 53.91 | 93.78 |
| NI16-1 | 24,585,025 | 7,309,651,400 | 52.84 | 91.46 |
| NI16-2 | 23,455,484 | 6,970,605,030 | 52.86 | 91.34 |
| NI16-3 | 25,867,254 | 7,696,402,230 | 53.57 | 92.42 |
| CS23-1 | 20,663,995 | 6,150,163,452 | 53.1 | 92.05 |
| CS23-2 | 21,293,847 | 6,347,181,232 | 53.55 | 92.32 |
| CS23-3 | 23,458,489 | 6,980,772,354 | 52.98 | 91.85 |
| NS23-1 | 20,935,773 | 6,233,681,180 | 52.97 | 92.03 |
| NS23-2 | 23,708,980 | 7,034,286,196 | 52.07 | 90.94 |
| NS23-3 | 20,171,914 | 5,997,708,982 | 52.86 | 91.84 |
| CI23-1 | 21,061,698 | 6,284,076,390 | 52.56 | 92.59 |
| CI23-2 | 22,227,801 | 6,606,763,540 | 52.53 | 91.72 |
| CI23-3 | 23,042,123 | 6,878,224,472 | 53.78 | 92.24 |
| NI23-1 | 22,212,352 | 6,606,848,126 | 52.55 | 91.38 |
| NI23-2 | 24,291,592 | 7,228,490,884 | 52.84 | 91.48 |
| NI23-3 | 21,096,478 | 6,299,035,518 | 53.29 | 92.52 |
| CS30-1 | 21,367,259 | 6,373,625,948 | 53.94 | 92.51 |
| CS30-2 | 19,616,420 | 5,854,269,718 | 53.87 | 93.2 |
| CS30-3 | 27,153,032 | 8,123,073,376 | 55.73 | 92.89 |
| NS30-1 | 27,458,777 | 8,197,107,106 | 54.73 | 92.17 |
| NS30-2 | 24,390,676 | 7,294,016,192 | 55.78 | 93.09 |
| NS30-3 | 20,275,615 | 6,063,454,278 | 55.95 | 93.52 |
| CI30-1 | 22,792,198 | 6,809,605,288 | 55.26 | 92.55 |
| CI30-2 | 20,645,068 | 6,172,715,352 | 53.36 | 93.35 |
| CI30-3 | 23,796,273 | 7,121,713,788 | 55.79 | 93.49 |
| NI30-1 | 20,038,060 | 5,996,283,102 | 56.07 | 93.93 |
| NI30-2 | 22,560,260 | 6,747,577,234 | 55.06 | 93.58 |
| NI30-3 | 22,862,181 | 6,830,720,850 | 55.77 | 92.87 |

Table S4 PacBio Sequel Full-Length Transcriptome Data evaluation table

| Sample ID | Platform | CCS Reads | FLNC Reads | HQ Isoforms | Consensus Isoforms | Mean CCS Length (bp) | FLNC (%) |
| --- | --- | --- | --- | --- | --- | --- | --- |
| Smooth bromegrass | PacBio Sequel | 560,075 | 526,768 | 170,341 | 170,387 | 1,645 | 94.05 |

Table S5 Numbers of DEGs in different modules

| Modules | DEGs number |
| --- | --- |
| black | 119 |
| blue | 6756 |
| brown | 878 |
| green | 244 |
| grey | 24 |
| magenta | 45 |
| pink | 89 |
| purple | 38 |
| red | 123 |
| turquoise | 10214 |
| yellow | 323 |

Table S6 Expression of gliadin related genes

| Transcripts ID | FPKM | | CI16_vs_NI16 | |
| --- | --- | --- | --- | --- |
|  | CI16 | NI16 | *P* value | Log_2_FC |
| *BiGli1*: P37_transcript_22930 | 182.2533333 | 3866.723333 | 3.26E-05 | 2.104497268 |
| *BiGli2*: P37_transcript_25445 | 566.9833333 | 5025.246667 | 1.38E-08 | 2.529933302 |

Table S7 Mean germination time and seedling fresh weight of transgenic Arabidopsis lines overexpressing *BiGli1* and *BiGli2*.

| Genotype | Mean Germination Time (Days) (Mean ± SD) | Change vs. WT (%) | Seedling Fresh Weight (g) (Mean ± SD) | Increase vs. WT (%) |
| --- | --- | --- | --- | --- |
| Col | 4.82 ± 0.04 | N/A | 0.0128 ± 0.0005 | N/A |
| *BiGli1*-OE2 | 4.70 ± 0.03 | -2.5 | 0.0162 ± 0.0003 | 26.9 |
| *BiGli1*-OE4 | 4.72 ± 0.01 | -2.1 | 0.0136 ± 0.0005 | 6.5 |
| *BiGli1*-OE6 | 4.80 ± 0.06 | -0.5 | 0.0127 ± 0.0008 | -0.9 |
| *BiGli2*-OE1 | 4.68 ± 0.04 | -3 | 0.0134 ± 0.0005 | 4.9 |
| BiGli2-OE3 | 4.74 ± 0.04 | -1.7 | 0.0141 ± 0.0002 | 9.9 |
| *BiGli2*-OE6 | 4.73 ± 0.06 | -1.9 | 0.0151 ± 0.0010 | 18 |
